# Enhancement of a nuclear factor of activated T cells (NFAT) reporter for the study of G protein-coupled receptors

**DOI:** 10.1038/s42003-026-10110-5

**Published:** 2026-04-26

**Authors:** Edward Wills, Anjana Saji, Jonathan Sumner, Aman Khan, Claudia M. Sisk, Mona Shehata, Benjamin Taylor, Graham Ladds

**Affiliations:** 1 Department of Pharmacology, University of Cambridge, Tennis Court Road, Cambridge, CB2 1PD UK; 2 Cell Immunology, Discovery Sciences, R&D, AstraZeneca, Cambridge, UK; 3 Bioassay, Biosafety and Impurities, BioPharmaceutical Development, AstraZeneca, Cambridge, UK

**Keywords:** Pharmacodynamics, Receptor pharmacology

## Abstract

Cell-based assays are fundamental to G protein-coupled receptor (GPCRs) drug discovery. As the field strives to increase the use of physiological cell types with endogenous receptor expression, enhancing the sensitivity of simple-to-use assays unlocks new screening modalities. Here, we enhanced the responsivity of a Nuclear Factor of Activated T cells response element (NFAT-RE) reporter, by concatenating the IL-2 promoter-derived triplicate-binding sites, to produce three nano-luciferase reporter constructs termed NFAT 2X, NFAT 3X and NFAT 4X. Our enhanced reporters demonstrate larger maximal Fold Induction (FI) when co-expressed with both primarily and secondarily Gα_q/11_-coupled GPCRs. This pattern was maintained when stimulating endogenous GPCRs in a panel of immortalised cell lines (HEK293, HeLa, A549, and HEK293T) and allowed us to observe Sphingosine-1-Phosphate (S1P)-mediated signalling in primary human CD8^+^ T cells via CRISPR/Cas9 knock-in. Our NFAT-reporter T cells demonstrate the reporters potential for use in bi-allelic expression systems and primary cell types.

## Abstract (web summary)

Cell-based assays are fundamental to G protein-coupled receptor (GPCRs) drug discovery. Here we report the enhancement of a Nuclear Factor of Activated T cells response element reporter for use in bi-allelic expression systems and primary cells.

## Introduction

**Fig. 1:**
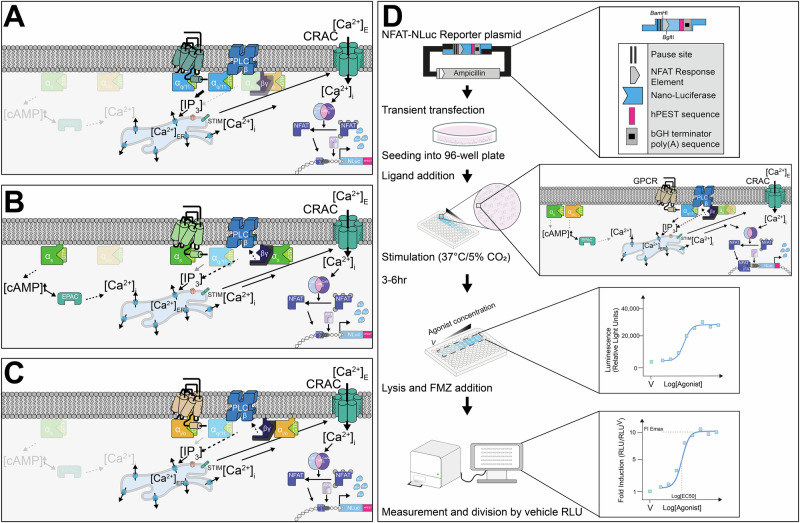
NFAT-RE induction occurs downstream of GPCR activation and is a simple plate-based assay system. **A** Simplified illustrative schematic of Gα_q/11-_ mediated NFAT activation. Primarily, Gα_q/11_-coupled GPCRs activate NFAT following Gα_q/11_-mediated stimulation of phospholipase Cβ s (PLC-β)s to produce IP_3_ and diacylglycerol (DAG). The IP_3_ activates IP_3_Rs in the endoplasmic reticulum (ER), causing Ca^2+^ release into the cytoplasm. Brief cytoplasmic Ca^2+^ spikes are prolonged by Ca^2+^ influx through CRAC channels, leading to the calmodulin-dependent activation of calcineurin (CaN). CaN dephosphorylates NFAT monomers, allowing them to dimerise or associate with partners such as AP-1 to induce transcription at NFAT-REs. By placing a nano-luciferase (NLuc) gene downstream of NFAT-REs, NFAT activation leads to NLuc transcription and translation. Both primarily Gα_s_- (**B**), and Gα_i/o_-coupled (**C**) GPCRs can activate NFAT signalling through secondary coupling to Gα_q/11_ (**B**,** C**), or via their Gβγ subunits, when primed by Gα_q/11_^11,12^. Gα_s_-coupled GPCRs, also mediate calcium signalling through the Exchange Protein Activated by cAMP (**B**; EPAC). **D** Schematic depicting stages of NFAT-RE reporter assay to detect GPCR signalling. Reporter cassettes consist of a pause site, genetic response element, minimal promoter, NLuc gene, PEST sequence and bovine growth hormone (bGH) poly(A) sequence. In-assay, GPCR agonists are used to elicit calcium signalling via Gα_q/11_ and other secondary pathways. Cells transfected with reporter produce NLuc (measured as relative light units) in a concentration-dependent manner to GPCR ligands. Responses can be baseline-corrected to the vehicle-treated cells (RLU/RLU^Vehicle^) to control for transfection efficiency and cell number.

Transcriptional response element (TRE) reporter assays offer simple and cost-effective observation of transcriptional events following G protein-coupled receptor (GPCR) activation^1–4^. Linking a TRE upstream of reporter genes such as nano-luciferase (NLuc) allows for the detection of transcription factor activity. The nuclear factor of activated T cells (NFAT) response element (NFAT-RE) can be used to indicate past changes in intracellular calcium ion (Ca^2+^) concentration ([Ca^2+^]_i_), elicited by GPCR signalling^5^. Although GPCR-dependent increases in [Ca^2+^]_i_ are predominantly incurred following Gα_q/11_ activation, contributions can be made downstream of Gα_s_ and Gα_i/o_ activation through their Gβγ subunits or other effectors^6–12^. Activated GTP-bound Gα_q/11_ subunits bind to phospholipase C-β (PLC-β) proteins^13^, promoting the hydrolysis of phosphatidylinositol-4,5-bisphosphate (PIP_2_) into diacylglycerol (DAG) and 1,4,5-inositol trisphosphate^14^ (IP_3_; Fig. 1A). Soluble IP_3_ binds and opens IP_3_ receptors (IP_3_R) in the endoplasmic reticulum (ER), releasing Ca^2+^ into the cytoplasm (Fig. 1A). Downstream of Gα_s_ activation, the second messenger species cyclic adenosine monophosphate (cAMP) can not only directly increase IP_3_R sensitivity^15–17^, but can also activate the exchange protein activated by cAMP 1 or 2 (EPAC1/2), leading to Ca^2+^ release^8–10,18^ (Fig. 1B). Furthermore, a direct mechanism for Gα_s_-Gβγ mediated Ca^2+^ mobilisation has recently been demonstrated^12^. Therein, Gα_s_-Gβγ activation of PLC-β2/3 was contingent on the presence of active Gα_q/11_ priming—with little influence of EPAC1/2 reported^12^. Previously, a similar requirement of active Gα_q/11_ priming for Gα_i_-Gβγ-mediated calcium responses was observed in a variety of cellular contexts^11^ (Fig. 1C). Ultimately, these pathways converge to cause Ca^2+^ efflux from the ER into the cytosol. Elevated cytosolic Ca^2+^ concentration is then sustained by stromal interaction molecule 1 (STIM1)-mediated opening of store-operated Ca^2+^ release-activated Ca^2+^ (CRAC) channels, further promoting calmodulin-dependent activation of the phosphatase calcineurin^19,20^ (CaN; Fig. 1A–C). Activated CaN dephosphorylates NFAT monomers, inducing their translocation to the nucleus where they can homodimerise^21^ or associate with partners such as activator protein 1 (AP-1)^22^, to induce transcription downstream of NFAT-REs^23,24^. Recently, Gα-null and specific Gα knockout HEK293 cells were used to demonstrate that although Gα_q/11_ is required for NFAT-RE induction by the angiotensin 1 receptor (AT_1_R) and cholecystokinin A receptor (CCK_1_), removal of other Gα subunits also reduced responsivity^25^.

In the genome, NFAT-REs exist as short sections of DNA embedded within the promoter for a particular gene, surrounded by other TREs such as the Nuclear factor kappa B (NfkB), the serum response element (SRE), and the cAMP response element (CRE). Although the first NFAT-RE sequence was discovered within the IL-2 promoter^26^, there are many variations of the core 5’-GGAAAA-3’ sequence present in a variety of different promoters^20^. Variants of these sequences have been extracted, manipulated, and paired with reporter genes over the last three decades to investigate NFAT signalling in different contexts^5,26–33^. Nine copies of the NFAT-RE from the IL-4 promoter (5′-TGGAAAATT-3′) has been coupled with firefly luciferase to develop NFAT-Luc transgenic mice for the investigation of calcineurin/NFAT signalling effects on cardiac hypertrophy^27^. Primary human T cells have been isolated and virally transduced with an NFAT-green fluorescent protein (NFAT-GFP) construct utilising 6 NFAT-RE’s (IL-2 origin) to enable observation and isolation of antigen-stimulated cells using flow cytometry^28^. More recently, a quadruplet NFAT-RE reporter, in combination with Enhanced Beetle luciferase (ELuc), was transduced into engineered Jurkat chimeric antigen receptor (CAR) T cells to analyse CAR activation^29^. Both T cell studies tested two NFAT-RE configurations (3 and 6^28^ or 4 and 6 repeating units^29^), reporting higher stimulation-induced transcription in reporters with more NFAT-REs, although the quadruple NFAT-RE reporter was selected for Jurkat engineering due to lower background signal. A widely used method for reducing reporter background signal is the incorporation of destabilising sequences such as human proline, glutamic acid, serine and threonine-rich (hPEST) sequences^34^. Such sequences are endogenously present on many short half-life transcription factors and target them for ubiquitination and proteasomal degradation^35–37^. Increasing the number of triplicate binding sites (3,6,9) in an IL-8 promoter-derived (5′-GGAATTTCC-3′) NFAT-RE-NLuc-hPEST reporter was demonstrated to increase responsivity to ionomycin stimulation of HEK293 cells^30^. However, observable induction from carbachol stimulation of the Gα_q/11_-coupled muscarinic acetylcholine receptor 3 (M_3_R) required transfection of NFAT1 and NFAT4, or knockdown of the calcium chelating protein apoptosis-linked gene 2 (ALG-2)^30^.

Despite this evidence suggesting increased sensitivity of IL-2-derived NFAT-RE reporters following concatenation of triplicate binding sites^28,29^, commonly available NFAT-RE reporter constructs and cell lines, contain one triplicate binding site^5^. Here, we concatenated the IL-2-derived triplicate NFAT-RE of a pNFAT-RE-NLuc-hPEST reporter up to four repeats (NFAT 1X, NFAT 2X, NFAT 3X and NFAT 4X) with a simple and fast molecular cloning method. The concatenated-NFAT reporters demonstrated greater raw luminescence and fold induction (FI) responses, while minimally affecting the potency (log[EC50]) of Gα_q/11_-coupled GPCR-ligand profiles in HEK293T cells. The FI metric is the ratio of ligand-stimulated luminescence to vehicle-treated luminescence and can be interpreted alongside raw luminescence results to inspect the responsivity of reporters. Enhanced NFAT reporters appear particularly useful to observe responses from GPCRs known to have secondary couplings to Gα_q/11_, showing similar increases in sensitivity with negligible effects on potency. The increased sensitivity of NFAT-RE concatenates was maintained when stimulating endogenously expressed GPCRs in four different cell lines. Lastly, site-directed CRISPR knock-in of a concatenated reporter allowed for detection of Sphingosine-1-Phosphate (S1P) signalling in primary human CD8^+^ T cells. The increased responsivity in a CRISPR knock-in controls for transfection variations and acts as proof-of-concept for the deployment of these reporters in primary cell studies.

## Results

### Concatenating NFAT-RE triplicate binding sites of an NFAT-NLuc-PEST reporter enhances NLuc expression and reporter response

**Fig. 2:**
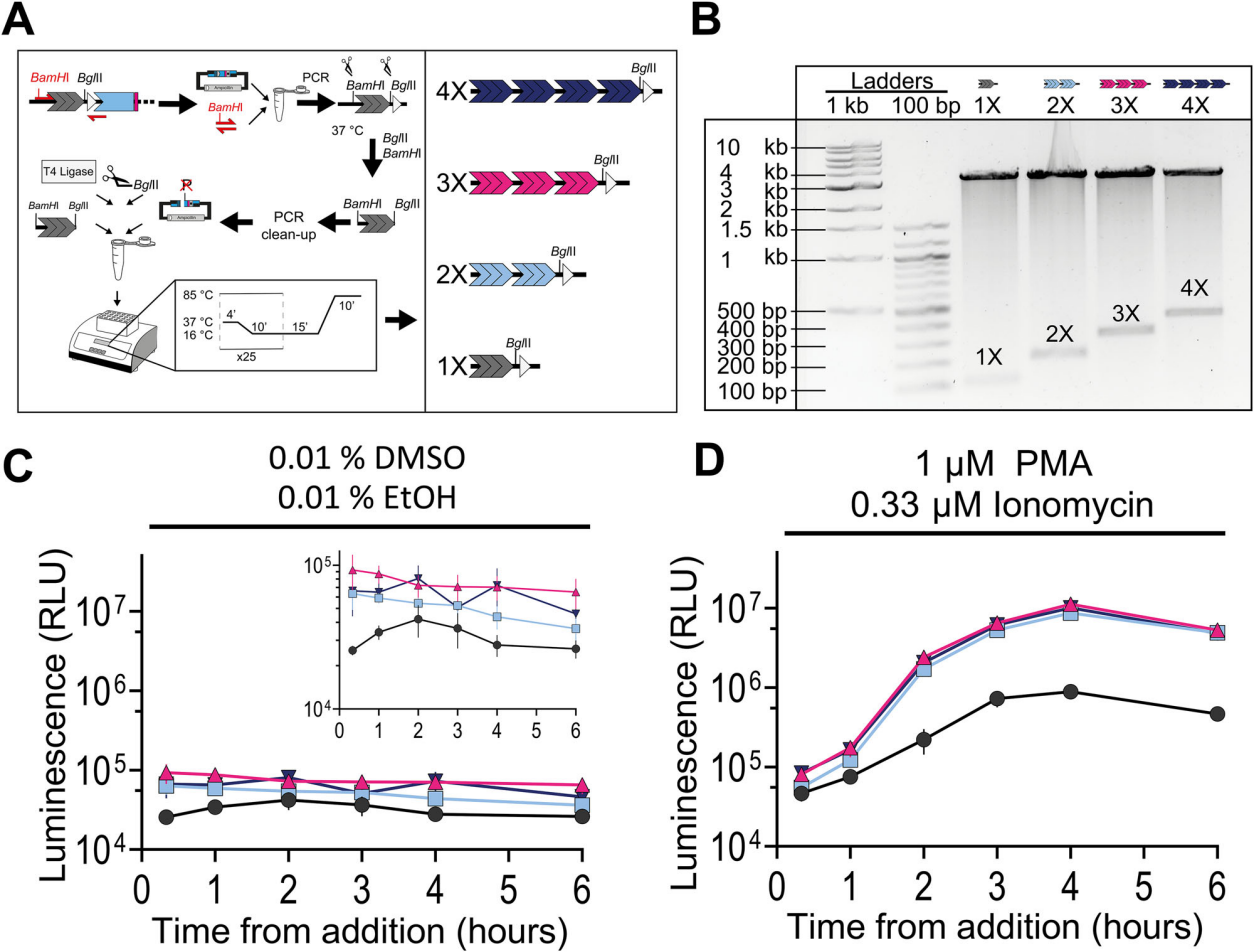
NFAT binding sites in a nano-luciferase reporter can be concatenated with a simple method to produce larger baseline NLuc expression and greater responses to calcium release in HEK293T cells. **A** Restriction enzymes *BamH*I and *Bgl*II create complementary cohesive ends, which, once ligated, are unrecognisable for both enzymes. We used this to concatenate NFAT-RE triplicate binding sites by PCR amplification of a *BamH*I-NFAT-RE-*Bgl*II sequence, digestion and then thermocycling. **B** This technique created three concatenated versions of NFAT-RE; NFAT 2X (211 bp), NFAT 3X (307 bp), NFAT 4X (403 bp), visualised on a 1% agarose w/v TAE gel, ran for 30 min at 120 V. **C** HEK293T cells were co-transfected with PTHR_1_ with either 1X (grey circles), 2X (blue squares), 3X (pink upward triangles) or 4X (purple downward triangles) NFAT-RE-NLuc-PEST reporter plasmids before stimulation with either vehicle (**C**; 0.01% DMSO, 0.01% EtOH; inset is rescaled axis to between 10^4^ and 10^5^ RLU) or (**D**) 0.33 μM of ionophore ionomycin and 1 μM PKC activator PMA for 6 h, with separate populations’ luciferase activity measured after 20 min,1, 2, 3, 4, and 6 h. The mean ± SEM (*n* = 3) of raw luminescence in relative light units (RLU), from three independent experiments performed in duplicate, is displayed on a logarithmic scale.

The TRE-reporter plasmids designed for this study consist of five elements (Fig. 1D): (1) a 5′ pause site to reduce baseline transcription, (2) the TRE selected with a minimal promoter, in this case the IL-2-derived NFAT-RE, (3) a reporter gene (NLuc), (4) a hPEST sequence, reducing baseline expression and increasing responsivity and (5) the bovine growth hormone (bGH) polyadenylation (poly(A)) sequence to terminate transcription. Populations of adherent cells can be transfected with NFAT-RE-NLuc-PEST constructs, seeded into a 96-well plate and assayed for a defined stimulation time with drugs at various concentrations (Fig. 1D). As raw relative light unit (RLU) responses can be influenced by day-to-day transfection efficiencies and may mask the concatenates’ larger baseline values, we utilise FI from vehicle to compare responsivity across reporters. We generate FI values by dividing each RLU value by the vehicle RLU (RLU/RLU^Vehicle^), to normalise for transfection efficiency and cell number. A key requirement for enhanced responsivity is that the basal luciferase production has not increased by a factor higher than the increase in maximal stimulated response, relative to the original reporter. Concatenate NFAT-RE constructs were generated by taking advantage of the compatible cohesive ends (CCEs) generated from *Bgl*II and *BamH*I digestion (Fig. 2A). The ligation of CCE’s generated from *Bgl*II and *BamH*I digestion eliminates the existence of either restriction site, allowing for recurrent digestion (at 37 °C) and ligation (at 16 °C) in a thermocycler. This concatenation produced NFAT-REs of 211 bp (2X), 307 bp (3X) and 403 bp (4X), visualisable with a *Nhe*I/*Hind*III digestion (Fig. 2B and Supplementary Fig. 1). When co-transfected with the Parathyroid 1 receptor (PTHR_1_) into HEK293T cells, concatenated constructs display larger unstimulated NFAT-based transcription over a 6-h window (Fig. 2C, inset). Moreover, when co-stimulated with commercially recommended NFAT positive controls, ionomycin and phorbol-12-myristate-13-acetate (PMA), concatenated-NFAT-REs produced RLU responses approximately tenfold higher than the original 1X construct when assayed between 2 and 6 h (Fig. 2D). Ionomycin acts as a Ca^2+^ ionophore, whilst PMA is a protein kinase C (PKC) activating ligand. Assay performance was evaluated by calculating the Z prime (Z’) factor across all timepoints for each reporter, yielding the highest Z’ factors from 2 to 4 h with NFAT 2X and NFAT 3X (Supplementary Fig. 2). As we observed the response to ionomycin/PMA began to plateau at 3 h stimulation (Fig. 2D), we performed all following NFAT-RE-based reporter assays with a 3-h stimulation time, to demonstrate a faster assay option for GPCR reporters.

Notably, similar concatenation of four other commercially available TRE’s did not increase responsivity to GPCR stimulation (Supplementary Fig. 3). In fact, concatenation of the quintuplet NfkB-RE decreased maximal fold induction and potency of PTHR_1_ responses in co-transfected (PTHR1: TRE-Reporter) HEK293T cells at 3 and 6 h (Supplementary Fig. 3A, B). Little effect on potency or fold induction was observed in HEK293T cells stably expressing the P2Y_12_ receptor (P2Y_12_R) when transfected with SRF-RE based reporter concatenates (Supplementary Fig. 3C), and minimal increases observed for an SRE reporter in the same context (Supplementary Fig. 3D). Lastly, increases in raw luminescence responses of concatenated CRE-duplet binding sites were observed in HEK293T cells stimulated with isoprenaline, but only translated to minimal increases in efficacy (FI Emax). Variations in maximal raw luminescence can be misleading when coinciding with increases in baseline expression, as responsivity may be unchanged or decreased (Supplementary Fig. 3A, E).

### Concatenate NFAT-NLuc-PEST reporters increases responsivity to primarily and secondarily Gα_q/11_-coupled GPCRs

**Fig. 3:**
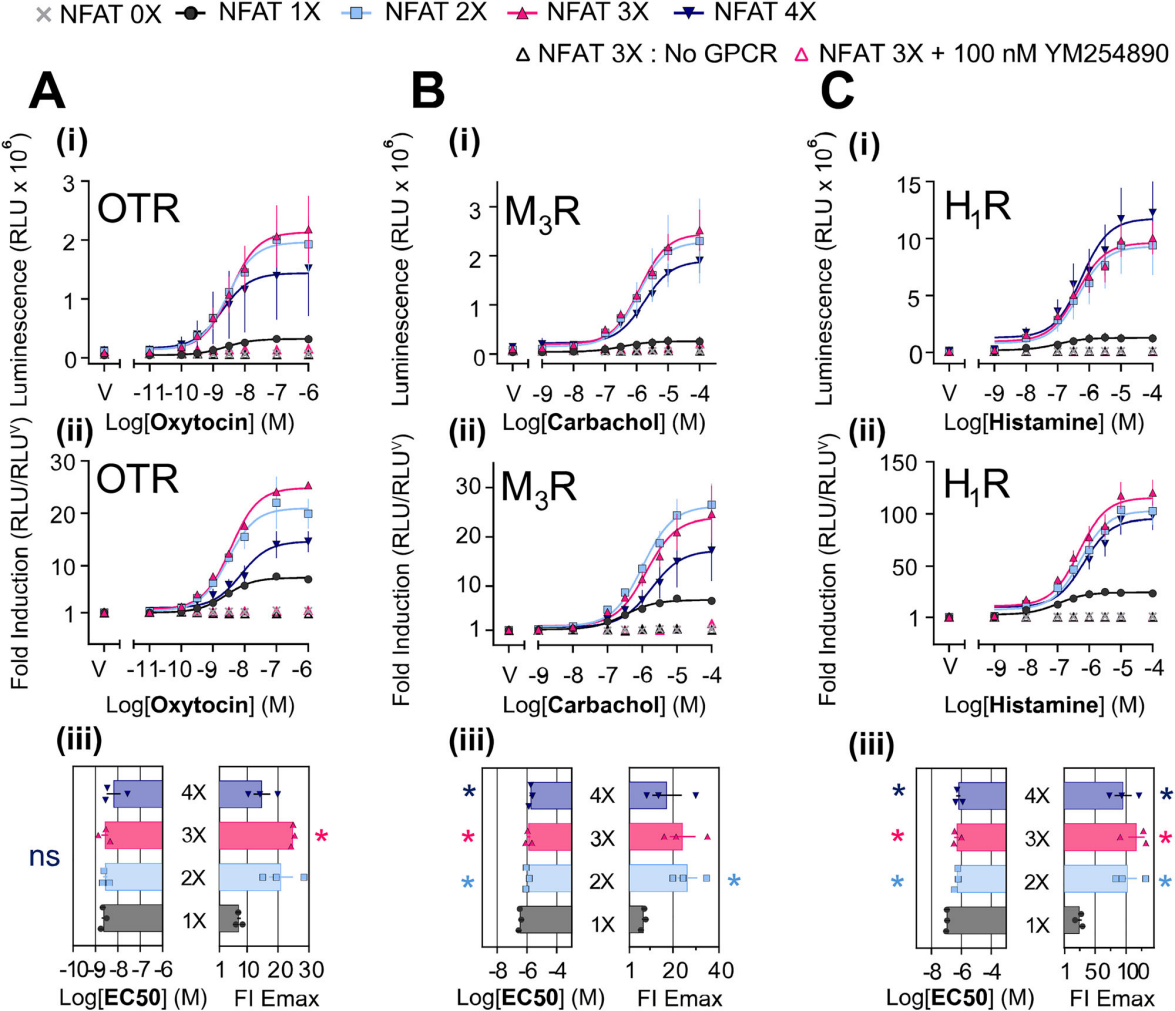
Concatenate NFAT-REs provide larger response range when measuring transfected primarily Gα_q/11_-coupled GPCRs in HEK293T cells. **A**–**C** Mean ± SEM (*n* = 3–5) luminescence responses, from HEK293T cells transfected with classically Gα_q_-coupled OTR (**A**), M_3_R (**B**) or H_1_R (**C**) in combination with pcDNA3.1(+) and either no NFAT (0X; grey crosses), 1X NFAT (grey circles), 2X NFAT (blue squares), 3X NFAT (pink upward triangles) or 4X NFAT (purple downward triangles) NFAT-RE-NLuc-PEST. Measured in relative light units (RLU) to increasing concentrations of oxytocin, carbachol, and histamine, respectively (**A**–**C**(i)). To compare responsivity, mean ± SEM fold induction (FI) responses were generated by dividing each RLU value by the vehicle (RLU/RLU^Vehicle^ (**A**–**C**(ii)). Responses (*n* = *3*) from cells transfected with NFAT 3X and empty vector rather than GPCR are shown with open black triangles (**A**–**C**(i–ii)). Responses (*n* = 2–3), (**A**–**C**(i–ii)) from cells co-transfected with NFAT 3X and GPCR but treated with 100 nM Gα_q/11_ blocker YM254890 are shown with open pink triangles. All concentration responses are fitted with three-parameter non-linear regression fits to obtain log[EC50] (M) and FI mean ± SEM maximal responses (FI Emax) (**A**–**C**(iii)). Datasets were compared statistically using one-way paired ANOVA with Fisher's least significant difference (LSD) test for planned comparisons of each concatenate vs the NFAT 1X reporter. Significance is reported as a coloured asterisk (*) when *p* < 0.05.

A simple way to study transcriptional endpoints of Gα_q/11_-coupled GPCRs is to transiently co-transfect them into HEK293T cells with an NFAT-RE reporter and observe their response to agonists. Here, co-transfection of three classically Gα_q/11_-coupled GPCRs with concatenated-NFAT-RE reporters was anticipated to elicit larger agonist-induced RLU and maximal FI responses without large changes in potency (Fig. 3A–C). Concatenating NFAT-REs produced larger RLU responses when co-transfected with the oxytocin receptor (OTR; Fig. 3A(i)), the M_3_R (Fig. 3B(i)), and the histamine 1 receptor (H_1_R; Fig. 3C(i)). This also translated to larger FI responses relative to vehicle-treated cells (Fig. 3A–C(ii)). When comparing the potency (log[EC50]) and efficacy (FI Emax) of oxytocin at the OTR, the NFAT 3X reporter was more responsive than 1X (Mean difference (MD) = 17.49 ± 0.55, 95% confidence interval (CI) [15.13, 19.85], *p* = 0.001, *n* = 3) whilst not changing potency (Fig. 3A(iii)). Similar increases in FI Emax were observed with the M_3_R (Fig. 3B(iii)) and the H_1_R (Fig. 3C(iii)). However, with small decreases in potency of carbachol (M_3_R NFAT 1X vs 3X log[EC50]: MD = -0.58 ± 0.07, 95% CI [−0.9, −0.25], *n* = 3, *p* = 0.016) and histamine (H_1_R NFAT 1X vs 3X log[EC50]: MD = -0.67 ± 0.14, 95% CI [−1.31, −0.24], *n* = 3, *p* = 0.047) at their respective GPCRs. Both the addition of 100 nM Gα_q/11_ inhibitor YM254890^38^ (YM) and the removal of NFAT binding sites (NFAT 0X; Fig. 3A–C; grey crosses) abolished the NFAT 3X responses for all three receptors (Fig. 3A–C(ii)). Thus, demonstrating the link between NFAT-RE induction and Gα_q/11_ activation, for these GPCR-ligand pairings in HEK293T cells—which were also confirmed to be GPCR-dependent by transfecting empty vector (Fig. 3A–C; black triangles). Amongst these GPCRs, the NFAT 3X and NFAT 2X reporters appeared the most responsive. When benchmarking the NFAT 3X construct against the NFAT 1X and a top-of-class commercially available reporter (pNL[NFAT-NLucP-Hygro] from Promega Corporation), all three reporters captured full agonist (carbachol) and partial agonist (pilocarpine) stimulation of the transfected M_3_R (Supplementary Fig. 4A, B). However, the NFAT 3X construct displayed a near 3-fold increase in responsivity for both agonists, when compared to the pNL[NFAT-NLucP-Hygro].

**Fig. 4:**
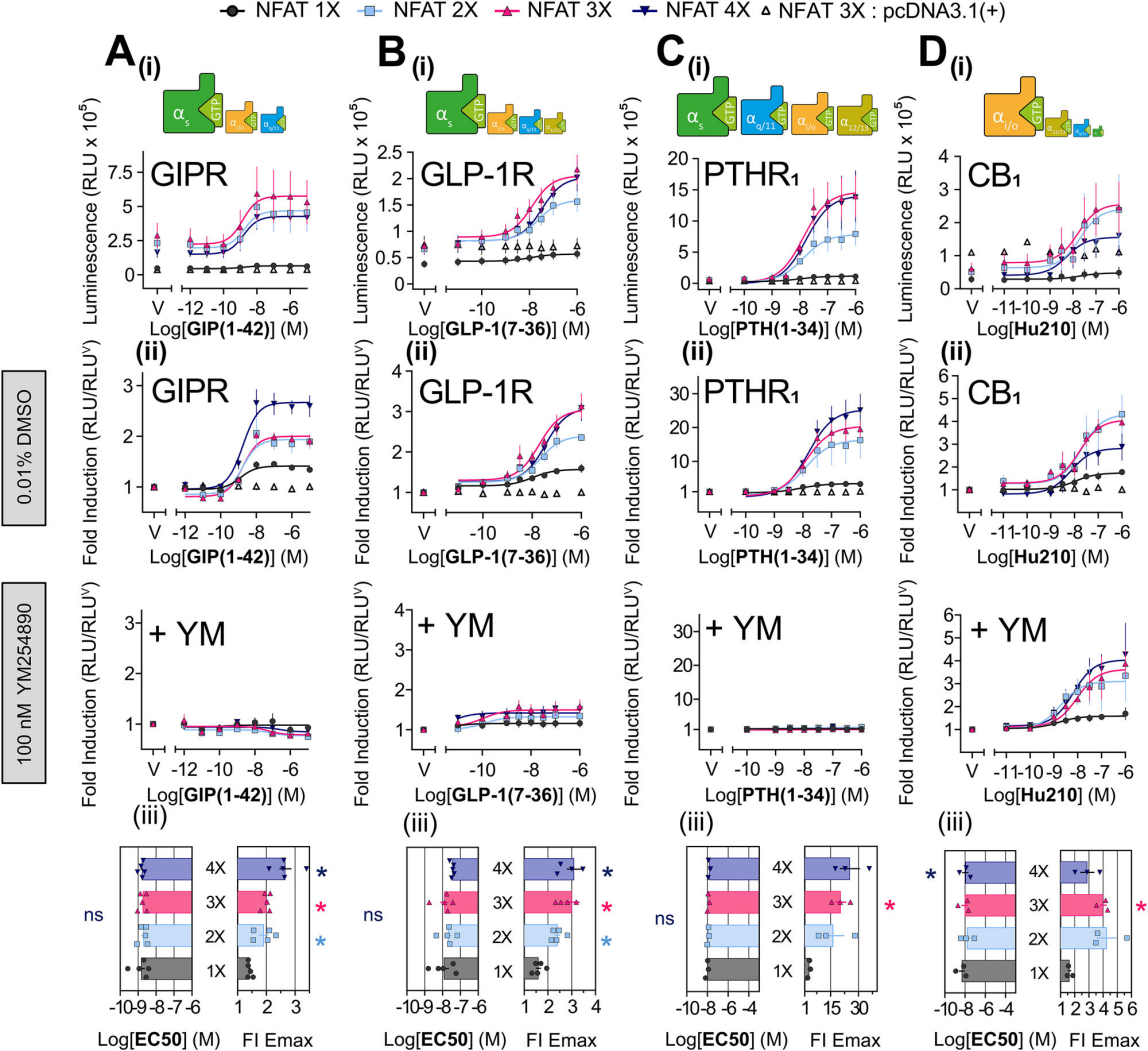
Concatenated NFAT-REs provide larger response range when measuring primarily Gα_s_ and Gα_i/o_ coupled GPCR signalling in HEK293T cells. **A**–**D** Mean ± SEM (*n* = 3–5) responses, from HEK293Ts transfected with primarily Gα_s_-coupled class B1 GPCR GIPR (**A**;*n* = 5), GLP-1R (**B**; *n* = 5), PTHR_1_ (**C**; *n* = 3) and primarily Gα_i_-coupled CB_1_R (D; *n* = 3) in combination with either 1X (grey circles), 2X (blue squares), 3X (pink upward triangles) or 4X (purple downward triangles) NFAT-RE-NLuc-PEST and pcDNA3.1(+). Proposed Gα couplings from G protein biosensor studies are illustrated for each GPCR^48^. Responses were measured in RLU to increasing concentrations of GIP(1–42) (**A**), GLP-1(7–36) (**B**), PTH(1–34) (**C**) and Hu210 (**D**), respectively (**A**–**D**(i)). To assess responsivity, mean ± SEM FI responses were generated by dividing each RLU value by the vehicle (RLU/RLU^Vehicle^ (**A**–**D**(ii)). Responses (*n* = 2–5) from cells transfected with NFAT 3X and empty vector rather than GPCR are shown with open black triangles (**A**–**C**(i–ii)). To assess contributions of Gα_q/11_ to responses (**A**–**D**(ii), lower panel, *n* = 3–5), cells were treated (including 30 min pretreatment) with 100 nM YM254890 to block activation. Concentration responses are fitted with three-parameter non-linear regression fits—from which mean ± SEM log[EC50] (M) and FI Emax can be extracted and compared (**A**–**D**(iii)). Datasets were compared statistically using one-way paired ANOVA with Fisher's least significant difference (LSD) test for planned comparisons of each concatenate vs the NFAT 1X reporter. Significance is reported as a coloured asterisk (*) when *p* < 0.05.

Although the signalling window enhancement of the NFAT-RE reporters is useful for studying primarily Gα_q/11_-coupled GPCRs, it could be instrumental for the study of NFAT activation by GPCRs which do not primarily couple to Gα_q/11_-PLC-β-Ca^2+^ (Fig. 1B, C). This can occur with GPCRs that exhibit minor Gα_q/11_ coupling alongside their primary G protein partner, but also through Gβγ subunits derived from their primary G protein, if PLC-β is primed by a Gα_q/11_^11,12^. The gastric inhibitory peptide receptor (GIPR) and glucagon-like-peptide 1 receptor (GLP-1R) are class B1 GPCRs targeted for the treatment of obesity and diabetes, with potential therapeutic benefits for Alzheimer’s and cardiovascular disease^39,40^. Moreover, they are known to exhibit secondary couplings to Gα_q/11_ and elicit intracellular Ca^2+^ mobilisation when expressed in HEK293 cells^41–44^. With the original NFAT-RE 1X sequence, GIPR (Fig. 4A) and GLP-1R (Fig. 4B) co-transfected cells have a small range with an FI Emax of 1.42 ± 0.04 FI (*n* = 5) and 1.60 ± 0.11 FI (*n* = 5), respectively (Fig. 4A, B(ii-iii)). Concatenate NFAT-RE reporters NFAT 4X and NFAT 3X significantly increase these FI’s to 2.66 ± 0.21 FI (GIPR, NFAT 4X, *p* = 0.0024, *n* = 5) and 3.01 ± 0.32 FI (GLP-1R, NFAT 3X, *p* = 0.013, *n* = 5), respectively. For GLP-1R, NFAT 4X achieves a similar FI (3.10 ± 0.32 FI), but reduces GLP-1(7–36) potency relative to the original reporter (NFAT 4X log[EC50]; −7.45 ± 0.05 vs NFAT 1X; −7.93 ± 0.28). When benchmarking the NFAT 1X and NFAT 3X reporters against the commercially available pNL[NFAT-NLucP-Hygro] using the GLP-1R, the NFAT 1X and pNL[NFAT-NLucP-Hygro] produced negligible responses, whilst NFAT 3X displayed a low-magnitude but pronounced concentration-response (Supplementary Fig. 5A, B). Inhibition of Gα_q/11_ with 100 nM YM fully attenuated the GIPR responses from all reporters (Fig. 4A(ii), lower panel), but only partially reduced GLP-1R NFAT 2X and NFAT 3X induction (Fig. 4B(ii), lower panel). Interestingly, the residual NFAT 3X response has a potency (log[EC50]: −9.48 ± 0.43 (M)) more akin to GLP-1R-mediated cAMP production^41^. The PTHR_1_ is another class B1 GPCR primarily coupled to Gα_s_, but can also activate Gα_q/11_, Gα_i/o_, and Gα_12/13_ proteins^45–47^. Here, we observed over ten-fold higher RLU responses from concatenates (Fig. 4C(i)), larger FI Emax (NFAT 3X Emax: 20.42 ± 2.93 FI and NFAT 4X Emax: 25.37 ± 5.43 FI, *n* = 3) compared to NFAT 1X (3.43 ± 0.43 FI, *n* = 3; Fig. 4C(ii)), and equipotency (Fig. 4C(iii)). Due to PTHR1 coupling promiscuity^48^, NFAT activation could be a convergent point for pathways downstream of multiple synergistic G protein sub-class activations. However, addition of YM entirely abolished the PTHR_1_ response to all reporters, demonstrating dependence on functional Gα_q/11_ (Fig. 4C(ii), lower panel). Lastly, we co-transfected our reporters with the Cannabinoid 1 receptor (CB_1_R)—a class A Gα_i/o_-coupled GPCR, reported to mediate Ca^2+^ signalling in specific cell types^49,50^. The NFAT 2X and NFAT 3X reporters provide enhanced RLU and FI responsivity when stimulating the CB_1_R with Hu210 (Fig. 4D(i–ii)). The NFAT 1X construct produced a 1.63 ± 0.12 FI Emax compared to 4.26 ± 0.71 (NFAT 2X; MD = 2.63 ± 0.79 95% CI [−0.77, 6.04], *p* = 0.08, *n* = 3) and 4.01 ± 0.24 (NFAT 3X; MD = 2.38 ± 0.79 95% CI [1.43, 3.30], *p* = 0.008, *n* = 3, Fig. 4D(iii)). Again, potency remained comparable across reporters (Fig. 4D(iii)). Interestingly, these responses were entirely YM resistant (Fig. 4D(iii)), suggesting a non-Gα_q/11-_ mediated pathway to NFAT activation.

### Increased NFAT-RE responsivity enhances observation of endogenous GPCR-NFAT signalling in four commonly used human cell lines

**Fig. 5:**
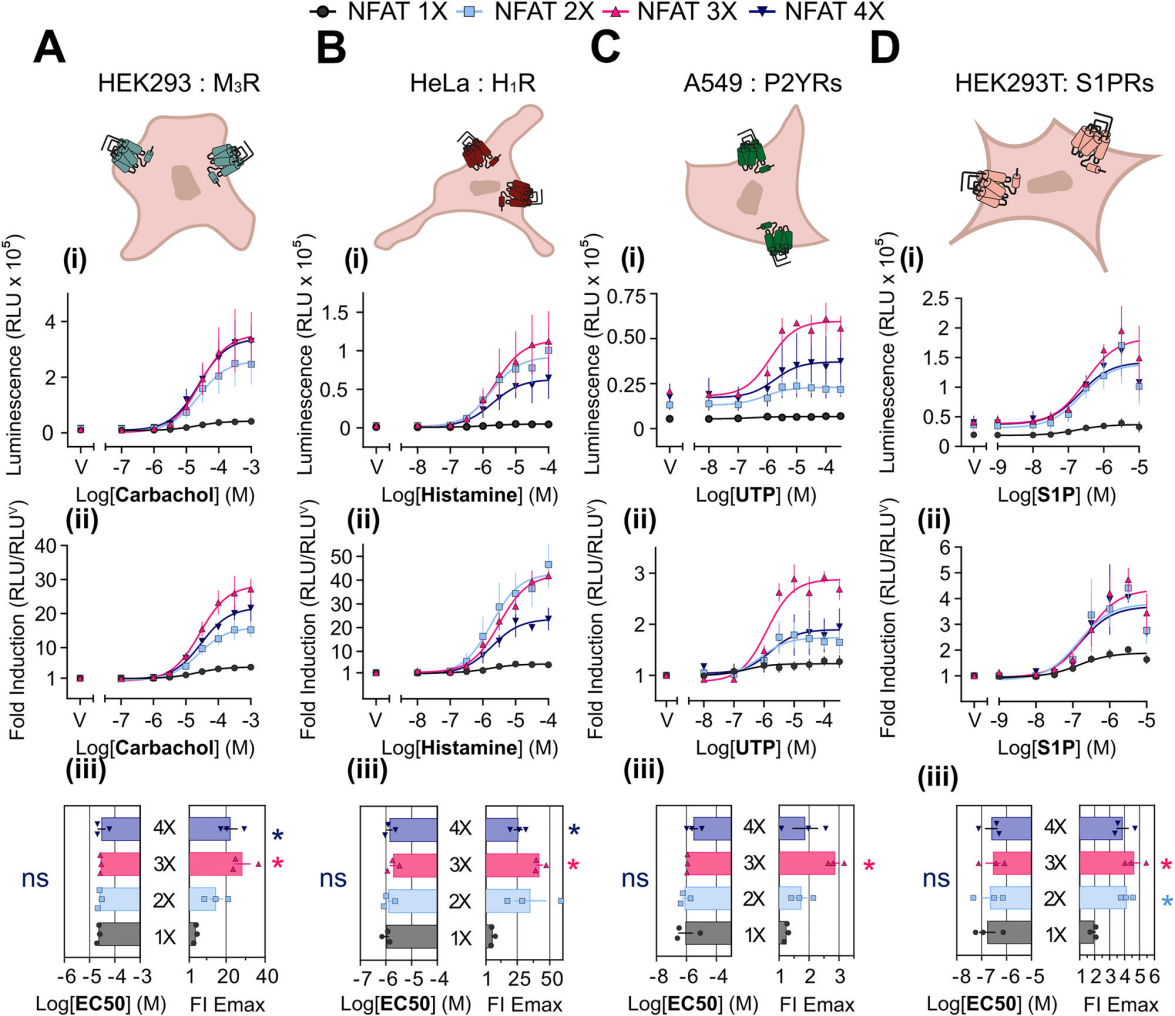
Concatenate NFAT-REs produce a larger response range when measuring endogenous GPCR-NFAT signalling in HEK293, HeLa, A549 and HEK293T cells. HEK293 (**A**; *n* = 3), HeLa (**B**; *n* = 4), A549 (**C**; *n* = 3) and HEK293T (**D**; *n* = 3) cells were transfected with either 1X (grey circles), 2X (blue squares), 3X (pink upward triangles) or 4X (purple downward triangles) NFAT-RE-NLuc-PEST (and pcDNA3.1(+) for HEK293T and HEK293s). Cells were stimulated with increasing concentrations of carbachol (**A**), histamine (**B**), uridine triphosphate (UTP) (**C**), and sphingosine-1-phosphate (S1P) (**D**) for 3 h before detection of luciferase activity, measured in RLU (**A**–**D**(i)). To assess responsivity, mean ± SEM FI responses were generated by dividing each RLU value by the vehicle (RLU/RLU^Vehicle^ (**A**–**D**(ii)). Concentration responses are fitted with a three-parameter non-linear regression fit—from which mean ± SEM log[EC50] (M) and FI Emax can be extracted and compared (**A**–**D**(iii)). Datasets were compared statistically using one-way paired ANOVA with Fisher's least significant difference (LSD) test for planned comparisons of each concatenate vs the NFAT 1X reporter. Significance is reported as a coloured asterisk (*) when *p* < 0.05.

As GPCR signalling outcomes can be dependent on both cellular background^51^ and receptor overexpression^52^, we wanted to determine whether increased responsivity translated to endogenous GPCR signalling in a variety of commonly used cell lines. Populations of HEK293, HeLa, A549 and HEK293T cells were transiently transfected with concatenated reporters and stimulated for 3 h before luciferase activity measurement. In HEK293 wild-type cells, we targeted endogenously expressed M_3_Rs using the muscarinic agonist carbachol—a benchmark Gα_q/11_ activation model (Fig. 5A(i–iii)). Cells transfected with the NFAT 1X reporter produced an FI Emax of 4.30 ± 0.51 FI with a potency of −4.65 ± 0.04 log[EC50] (M) (*n* = 3). NFAT 3X cells produced an over six-fold increase in FI max (28.34 ± 4.15, MD = 24.04 ± 4.63, 95% CI [4.12, 43.96], *p* = 0.035, *n* = 3), whilst maintaining similar carbachol potency (NFAT 3X log[EC50]; −4.57 ± 0.01 M, *n* = 3). Benchmarking the NFAT 1X and NFAT 3X against the commercially available pNL[NFAT-NLucP-Hygro] in HEK293 WT cells demonstrated NFAT 3X as a highly responsive reporter (Supplementary Fig. 6A–D). All three reporters produced concentration-responses to carbachol, but the NFAT 3X FI Emax was over 25-fold higher than the commercially available reporter (Supplementary Fig. 6C). Interestingly, pilocarpine (partial agonist) responses were not detectable with the endogenous M_3_R expression levels in HEK293 WT with the NFAT 1X and pNL[NFAT-NLucP-Hygro] reporters. However, a low-magnitude concentration response was observed with NFAT 3X (Supplementary Fig. 6B, D). Similarly to the endo-M_3_R in HEK293 WT cells, the concatenates enhance maximal RLU and FI in another benchmark Gα_q/11_ activation model—the endogenous H_1_R in HeLa cells (Fig. 5B(i–iii). Here, NFAT 3X showed the highest FI max (NFAT 3X; 42.00 ± 2.63 FI, vs NFAT 1X; 6.14 ± 1.02 FI, MD = 35.86 ± 3.31, 95% CI [21.62, 50.10], *p* = 0.0084, *n* = 3), with minimal effect on histamine potency (log[EC50]: NFAT 3X; -5.98 ± 0.09 vs NFAT 1X; -5.72 ± 0.14, MD = -0.26 ± 0.06, 95% CI [−0.53, 0.01], *p* = 0.053, *n* = 3; Fig. 5B(iii)). For our third endogenous signalling model, we stimulated A549 lung carcinoma cells with uridine triphosphate (UTP) to activate purinergic Y receptors (P2YRs; Fig. 5C). Here, the response from NFAT 1X transfected cells could be small enough to be disregarded (1.25 ± 0.04 FI, *n* = 3), compared to the substantial concentration-response when transfecting NFAT 3X (2.88 ± 0.16 FI, *n* = 3; Fig. 5C(ii)). Detectable endogenous M_3_R expression is lost in HEK293T cells (measured with NFAT 3X; Fig. 3B), compared to HEK293 wild-type cells (Fig. 5A). Therefore, we wanted to detect NFAT signalling mediated by other endogenously expressed GPCRs in HEK293Ts. Sphingosine-1-Phosphate (S1P) is a membrane-derived lysophospholipid with five human subtypes of GPCRs (S1PR_1-5_). Although none of these subtypes primarily couple to Gα_q/11_, there are reports of auxiliary Gα_q/11_ coupling for S1PR_2_ and S1PR_3_^53^ and pertussis toxin (PTX)-sensitive Ca^2+  ^  signalling (Gα_i/o_) for the other subtypes^54–56^. Moreover, S1PR_1_, S1PR_2_ and S1PR_3_ receptors are ubiquitously expressed in tissues^55^. Stimulation of HEK293T cells transfected with our concatenate reporters showed notable (FI Max: NFAT 2X; 4.14 ± 0.24 FI, NFAT 3X; 4.64 ± 0.42 FI, NFAT 4X; 3.87 ± 0.42 FI, *n* = 3) NFAT responses to S1P, exceeding the NFAT 1X transfected cells (FI max: 1.95 ± 0.13 FI, *n* = 3) with no meaningful changes to potency (log[EC50]: NFAT 1X; −6.78 ± 0.33, NFAT 2X; −6.66 ± 0.35, NFAT 3X; −6.55 ± 0.31, NFAT 4X; −6.61 ± 0.26, *n* = 3) (Fig. 5D).

Activation of NFAT TFs was attenuated by Gα_q/11_ blockade when stimulating both primarily Gα_q/11_- and Gα_s_-linked GPCRs (Figs. 3 and 4), suggesting Gα_q/11_-dependant signalling to NFAT TFs in these contexts. In the case of the Gα_s_-linked GPCRs, Gα_q/11_ dependency may be due to promiscuous direct Gα_q/11_ coupling of the GIPR, GLP-1R and PTHR_1_^43,45–47,57^, or Gα_q/11_ dependent of signalling from Gβγ subunits^11,12^. Surprisingly, however, the primarily Gα_i/o_-linked CB_1_R induced NFAT signalling in an entirely YM-resistant manner (Fig. 4D(ii)). Next, we deployed an array of chemical inhibitors to probe for other mediators of CB_1_R-NFAT signalling. PTX was used to eliminate Gα_i/o_ contributions, Rhosin to block RhoA signalling, and YM to investigate any synergistic effects of Gα_q/11_. Both Rhosin, PTX, and PTX with YM partially reduced basal luminescence and responsivity of the CB_1_R, to a similar degree (Supplementary Fig. 7). The YM addition to PTX treatment had no additive effect, however the addition of Rhosin with both PTX and YM produced the greatest reduction in basal signalling and Hu210 potency (Supplementary Fig. 7A, B; green triangles). As our investigations into non primarily Gα_q/11_-coupled GPCRs had been limited to GPCR overexpression contexts, we also wanted to probe the S1P-mediated NFAT responses in HEK293T cells (Fig. 5D and Supplementary Fig. 7C, D), which is likely downstream of primarily Gα_i/o_—linked S1PR_2_ and S1PR_3_^58^. Here, all treatments appeared to reduce both maximal luminescence (Supplementary Fig. 7C) and responsivity (Supplementary Fig. 7D) in the following rank order: PTX and YM < Rhosin < Rhosin, PTX and YM < overexpression of the IP_3_ kinase C (IP_3_KC). For context, the overexpressed IP_3_KC constitutively phosphorylates IP_3_ to inositol 1,3,4,5-tetrakisphosphate (IP_4_), eliminating IP_3_-mediated signalling^59^. These results suggest that the pathways from these Gα_i/o_-linked GPCRs to NFAT induction, in these cell contexts, are downstream of multiple mediators.

### Detection of S1P signalling in primary human CD8 ^+^ T cells using CRISPR knock-in requires concatenate NFAT-RE reporter

Concatenate NFAT-RE plasmids display enhanced responsivity when transfected into commonly used cell lines. However, we wanted to investigate whether they could unlock the ability to detect GPCR-NFAT signalling in primary human CD8 ^+^ T cells after bi-allelic CRISPR knock-in. Also, the targeted nature of a knock-in controls for any potential overexpression interference occurring in transient transfections of cell lines. NFAT signalling leads to the expression of various immuno-regulatory genes in T cells, such as IFN-gamma, IL-2, IL-4 and IL-8^23,24,28,29,60^ but may also play roles in CD8^+^ T cell exhaustion^61^. Although non-chemokine GPCR-mediated regulation of T cells has been understudied, recent work has highlighted Gα_s_-coupled GPCRs as both modulators of T cell dysfunction in cancer and limiting factors for immunotherapy of solid tumours^62^.

**Fig. 6:**
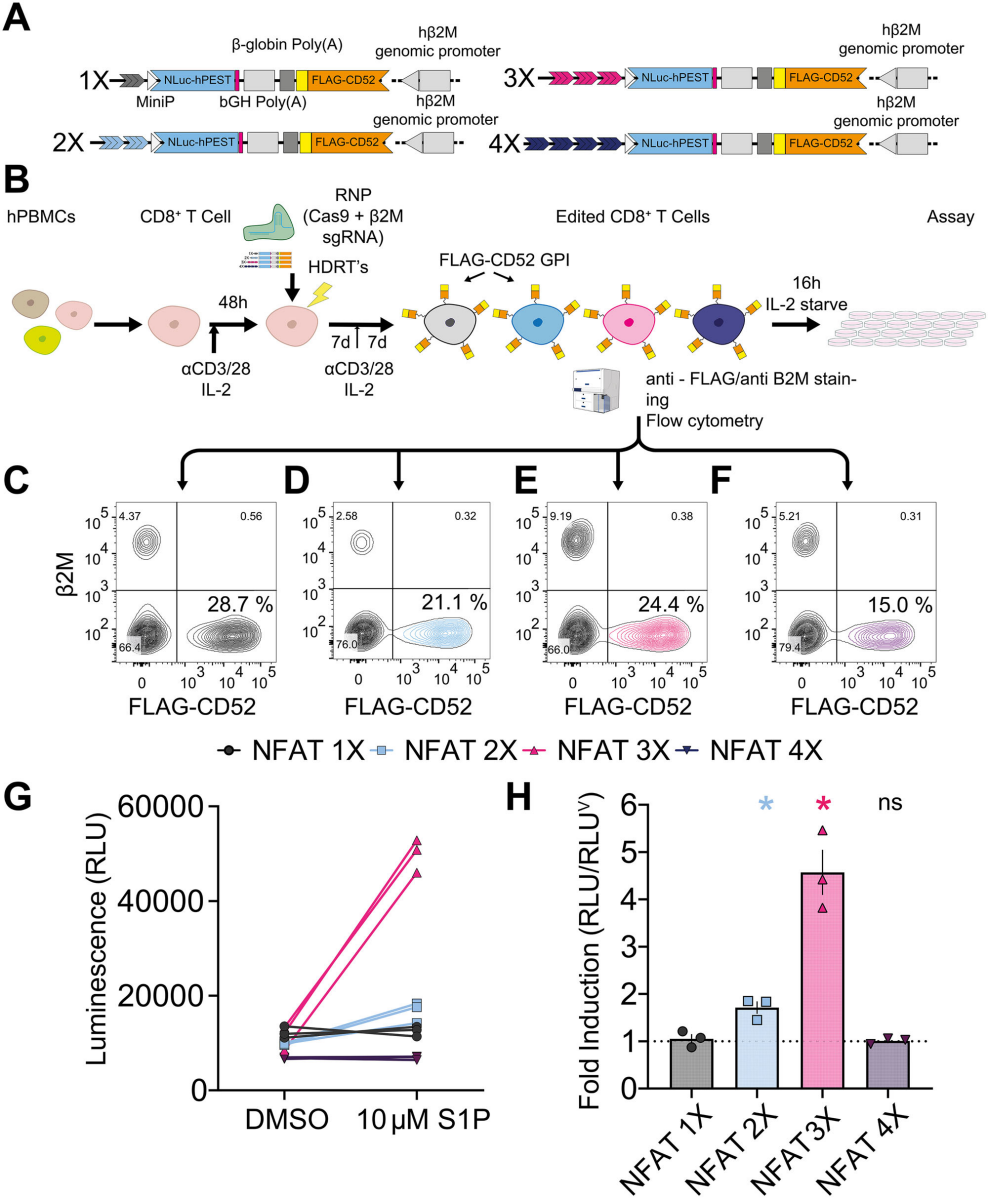
Concatenate NFAT 2X and NFAT 3X-NLuc reporters can detect sphingosine-1-phosphate signalling in primary human CD8 ^+^ T cells. **A** Concatenate NFAT-RE homology directed repair template (HDRTs) were designed to incorporate the reporter and FLAG-tagged, GPI-Anchored, CD52 gene expressed off the endogenous β-2-microglobulin promotor. **B** Human peripheral blood mononuclear cells (hPBMCs) are sorted to obtain CD8 ^+^ T cells from a single donor. These cells are stimulated with αCD3/28 and IL-2 to initiate proliferation, before nucleofection with ribonuclear protein (RNP) and HDRTs. Editing efficiency of each NFAT knock-in (NFAT-KI) population is determined via flow cytometry of anti-2M and anti-FLAG-stained cells (**C**–**F**), where the FLAG-CD52^+^ and β2M (−) population of cells are considered successfully edited. Cells are then left to recover for 2-weeks before 18-h IL-2 starvation followed by S1P stimulation for 4 h. **G** Mean luminescence, in relative light units (RLU), from three independent experiments performed in duplicate of NFAT 1X-KI (grey circles), NFAT 2X-KI (blue squares), NFAT 3X-KI (pink upward triangles) or NFAT 4X-KI (purple downward triangles) cells treated with 0.1% DMSO (vehicle) or 10 μM sphingosine-1-phosphate. To assess responsivity, the mean ± SEM fold induction (FI) responses were generated by dividing the S1P- treated cells' RLU value by the vehicle (RLU/RLU^Vehicle^). Datasets were compared statistically using one-way paired ANOVA with Fisher's least significant difference (LSD) test for planned comparisons of each concatenate vs the NFAT 1X reporter. Significance is reported as a coloured asterisk (*) when *p* < 0.05.

We designed a CRISPR knock-in construct with a Homology Directed Repair Template (HDRT) containing an interchangeable TRE region, minimal promoter, NLuc-PEST, and bgH poly(A) for the reporter (Fig. 6A). The HDRT also contained a coding DNA sequence (CDS) for a FLAG-tagged CD52 antigen, which becomes glycosylphosphatidylinositol (GPI)-anchored to the membrane of successfully edited cells, after insertion downstream of the β2-microglobulin (β2M) promoter using β2M guide RNAs (gRNAs). From other studies^62^, we were confident of a Gα_s_-linked GPCR response to adenosine via the adenosine A_2A_ receptor in T cells. Therefore, we validated our editing strategy and ability to detect GPCR signalling using a highly responsive CRE-NLuc-PEST knock-in reporter (Supplementary Fig. 8A). After confirming functionality in HEK293T cells (Supplementary Fig. 8B), T cells were edited, and FLAG-CD52 KI populations confirmed (Supplementary Figs. 8C–E and 9). The CRE-NLuc-PEST-KI population demonstrated concentration-dependent NLuc expression when stimulated with forskolin, adenosine, and, to a lesser extent, adrenaline (Supplementary Fig. 8F). To develop concatenated-NFAT-RE-NLuc-PEST-KI cells, populations of CD8^+^ T cells were activated by αCD3/28 and IL-2 supplementation to stimulate proliferation before editing via nucleofection (Fig. 6B). Editing efficiency was determined by flow cytometry of anti-FLAG and anti-β2M-stained cells (Fig. 6C–F). Efficiencies were comparable across NFAT 1X (28.7%), NFAT 2X (21.1%) and NFAT 3X (24.4%) but reduced with NFAT 4X (15%). Equal numbers (scaled by KI-efficiency) of FLAG-CD52^+^ cells from each population were IL-2 starved to reduce background NFAT activation. When stimulated with S1P, the NFAT 1X-KI and NFAT 4X-KI cells showed no difference to dimethylsulfoxide (DMSO; vehicle) treated cells (Fig. 6G), whereas increases in luminescence and FI (Fig. 6G, H) were observed for NFAT 2X-KI (1.71 ± 0.13 FI, *n* = 3) and NFAT 3X-KI (4.57 ± 0.48 FI, *n* = 3) cells. This cross validated our results from overexpression systems in primary cells with a reporter copy number of two.

## Discussion

To enact longer-term cellular changes, GPCRs alter the transcription of target genes through TREs such as NFAT-RE. Although TRE assays are long since established, their detection of downstream signalling events, amenability to high-throughput screening, and affordability retain them as useful tools in pharmacology^1–4^. With regards to enhancing NFAT-RE reporters, multiple studies have presented evidence suggesting the benefits of concatenating the NFAT binding sites^28–30^ - mostly for studying T cell activation. These benefits appear independent of NFAT sequence, and gene promoter origin. Most notably, a pseudo-palindromic 3X IL-8 NFAT-RE was developed and shown to increase responsivity in specific conditions compared to a 1X version^30^. However, the requirement for ALG-2 knock down or NFAT TF overexpression to observe still relatively low FI M_3_R signalling in HEK293 cells suggests the IL-2 based sequence may be more suited for GPCR investigation.

Generally, most reporter assays in the literature stimulate from 6 up to 48 h^2,3,30,63,64^. Incorporation of a hPEST sequence on reporter luciferases is known to increase both the rate and magnitude of luciferase FI from vehicle-treated cells, when sensing GPCR signalling^5^. Combining this with the concatenates’ increased responsivity allowed for the reduction of assay duration. Thus, we tested our enhanced NFAT-RE reporters’ capability with 3-h stimulation times, as a more rapid reporter assay. When measuring GPCR signalling, long stimulation times have previously been suggested as a limitation of TRE-reporter assays, as GPCRs may become desensitised early in this period, leading to reduced responses^65^. With strongly NFAT-activating ligand-GPCR-cell relationships, such as endo-H_1_R in HeLa cells, transfected H_1_R or PTHR1 in HEK293T cells, and M_3_R in HEK293 cells, stimulation times could perhaps be reduced to 2 hs.

Concatenating IL-2-derived NFAT-RE triplicate binding sites is a simple yet effective method of increasing reporter responsivity. Responsivity was evaluated by calculating the FI from vehicle responses and interpreted alongside luminescence, as larger raw luminescence alone may not correlate to higher responsivity, as observed with NFAT 4X and the commercially available reporter construct (Supplementary Figs. 4–6). Reporting of both measures allows for distinction between genuine effects on ligand-induced responsivity and a general luciferase production increase. Concatenation of TRE sequences consistently increased basal reporter production but did not always amplify the maximal ligand-induced response to a higher degree—a criterion required for increased responsivity. Incidence of this can be observed with the alternative commercially available GPCR-related TREs (CRE/SRE/SRF-RE/NfkB), which did not yield the same increases in responsivity (Supplementary Fig. 3A–DE). The SRE and SRF-RE concatenate reporters demonstrated equal increases in both basal and maximal luminescence, leading to similar responsivity between concatenates. Meanwhile, CRE and NfkB concatenate FI was lower than the original constructs, due to larger increases in basal than in maximal luminescence production. Therefore, the NFAT-RE appears unique within this subset of TRE’s, perhaps due to stoichiometric differences in transcription factor binding between concatenated duplet, triplet and quintuplet binding sites.

When transiently transfecting classically Gα_q/11_ coupled GPCRs (M_3_R, H_1_R and OXTR), the increase of response from concatenates (particularly NFAT 3X) relative to NFAT 1X was outstanding (Fig. 3A). However, it must be noted that each of these receptors still produced robust responses with the original NFAT 1X construct. Also, when optimising signalling assay ranges, it is important to be wary of creating artificial potency changes due to maximising assay output (in this case, NLuc transcription). Although small, decreases in potency for concatenated reporters with the M_3_R and H_1_R in HEK23Ts were statistically significant, so should be highlighted. The largest change in potency is from the highest responding GPCR (H_1_R), suggesting the system reaches maximum NLuc production. Alternatively, apart from NFAT 4X GLP-1R, no changes in potency were observed with GPCRs not primarily coupled to Gα_q/11_ (Fig. 4A–D(iii)). Also, as NFAT signalling is generally less pronounced for these GPCRs (excluding PTHR_1_), the use of concatenate NFAT-RE’s generates responses over two-fold from vehicle. This enhanced range allows for potential antagonism studies at these receptors or efficacy differences between agonists and by benchmarking the NFAT 3X against a commercially available reporter, we demonstrate its potential utility for detecting previously unreported NFAT signalling. Moreover, increased RLU baselines could facilitate inverse agonist screening, for GPCRs that display constitutive activity.

Enhanced NFAT responses were shown across four commonly used cell lines, by stimulating endogenously expressed GPCRs. The concatenated reporters are applicable to various cellular contexts and do not require receptor overexpression. Of note, the observation of clear purinergic NFAT signalling in A549 cells was dependent on the expression of 2X, 3X or 4X NFAT-RE reporters. Intracellular Ca^2+^ responses have previously been detected in response to UTP stimulation of A549 cells^66,67^. There are four purinergic receptors that are activated by UTP (P2Y_2_, P2Y_4_, P2Y_6_ and P2Y_11_), which all couple primarily to Gα_q/11_ (IUPHAR GtoPdb)^68^. Most studies report P2Y_2_ co-expression with P2Y_6_ in A549 cells^69,70^, alongside the human protein atlas (HPA; https://www.proteinatlas.org/). The micromolar potency range of UTP in our NFAT-reporter system is comparable with the UTP potency reported in both an A549 proliferation assay^70^ and an intracellular Ca^2+^ assay^66^. Our NFAT reporters allow for detection of this signalling using simple transient transfection methods, targeting a cell type previously discussed to be ‘harder-to-transfect’ than HEK293T, HEK293 and HeLa cells^71^. Although transient transfections can be compared when performed in parallel on the same cells, developing a clonal concatenated-NFAT-RE-NLuc-PEST reporter cell line will reduce variability when studying specific GPCR signalling events or comparing multiple ligands. We opted not to use a cytomegalovirus (CMV)-luciferase transfection control, due to its inducibility by GPCR stimulation^72^. Though, the assay precision across days may be further optimisable through luciferase co-expression from a non-inducible promoter.

Across most GPCR and cell-type contexts, the NFAT 2X and NFAT 3X reporters display the highest responsivity in assays. Increases in responsivity did not correlate linearly with NFAT binding site number. The NFAT 4X reporter only outperformed others in two instances (GIPR and PTHR_1_) across all assays. The observed non-linear relationship may result from steric hindrance of TF binding, leading to a saturation of responsivity to GPCR signalling. Alternatively, the TF binding sites may be in excess of the population of activated TF complexes, when the sensor is transiently transfected. The path upstream of TRE induction involves multiple stages of signal amplification, crosstalk between signalling pathways, and the integration of events over time. We have observed small Gα_q/11_, Gα_i/o_ and RhoA independent NFAT signalling from the CB_1_R and after S1P stimulation in HEK293T cells. However, the S1P-mediated NFAT-RE induction was dependent on IP_3_ signalling (Supplementary Fig. 7C, D). Recently, GPCR-NFAT responses have been shown to be largely downstream of Gα_q/11_ signalling, but with input from all other G protein families^25^. As Gα_i/o_ coupled GPCRs, it was surprising that NFAT signalling was resistant to both Gα_i/o_ and Gα_q/11_ blockade, also given their reported interdependence for calcium signalling^11^. This suggests either Gα_s_ signalling crosstalk to Ca^2+^ mobilisation, Gα_z_ or Gα_z_-derived Gβγ interactions, or non-RhoA mediated Gα_12/13_ signalling. In the specific case of S1P signalling in HEK293T cells, S1P may be acting through either the S1PR_2_, S1PR_3_^58^ or both. Although usually described in the literature to be coupled to Gα_i/o_, Gα_q/11_ and Gα_12/13_^53,73^ there has been a report of chimeric-Gα_s_ coupling in the transforming growth factor-α (TGF-α) shedding assay^45,48^. Such coupling could result in Gα_s_ signalling from S1PR_2_ mediated crosstalk to NFAT activation, which would not have been inhibited by YM, PTX or Rhosin but attenuated with the transfection of IP_3_KC. Further investigation into how these traditionally Gα_i/o_-coupled GPCRs mediate NFAT signalling with a suite of molecular biosensors could identify the specific mechanisms in HEK293T cells. Due to TRE’s distal nature, they are not optimal sensors for the mechanistic determination of a signalling pathway. Rather, they are useful high-throughput screening tools to identify signalling before more proximal molecular investigations. To this end, our enhanced NFAT reporters may increase the sensitivity of reporter screening assays and even allow for the observation of previously unreported signalling, with non-canonical signalling routes.

We observed enhanced NFAT signalling sensitivity with our NFAT 2X and NFAT 3X sequences in primary CD8^+^ T cells (Fig. 6). The concatenated-NFAT edited cells facilitated the observation of S1P-mediated NFAT activation, whereas the original construct showed no fold induction compared to vehicle. Through interaction with immune cell S1PRs, S1P gradients ([S1P]_blood_ > [S1P]_lymph_ > [S1P]_interstitial_) orchestrate lymphocyte tissue distribution^74^ and play complex roles in anti-tumour immune responses. Knockdown of S1P transporter spinster homologue 2 (*SPNS2*) perturbed such gradients in a mice model of pulmonary metastasis. Concurrently reducing metastasis by inducing circulatory lymphopenia and concentrating natural killer (NK) and CD8^+^ T cells in the lung^75^. Alternatively, S1P-related lymphocyte sequestration is detrimental in glioblastoma pathology. S1PR_1_ down-regulation occurs in naïve T cells of glioblastoma patients, contributing to bone-marrow sequestration and lymphopenia^76^. Interestingly, S1PR_3_ expression in CAR-T cells has been associated with CAR-T cell exhaustion, and antagonism leads to better immunotherapy results and more recruitment of CD8^+^ T cells to the tumour environment^77^. Our enhanced NFAT reporters could be used to further study S1P interactions with such immunotherapies. Lastly, as the cAMP-PKA-CRE signalling axis has also been highlighted to suppress CAR-T cell immunotherapy^62^, the development of dual NFAT and CRE reporter CAR-T cells may be valuable for future investigations into GPCR interactions. To summarise, we have validated our enhanced NFAT reporters to study GPCR activation in a panel of GPCRs with different signalling preferences. We have also confirmed their enhancement when studying endogenously expressed receptors in both model and primary cells. These tools will aid researchers to develop further methods or to study specific ligand-GPCR-cell type relationships.

## Methods

### Molecular biology

Plasmid constructs consisting of a pause site, minimal promoter, luciferase (nano-luciferase, firefly luciferase, *Renilla* luciferase or *Green Renilla* luciferase), PEST sequence and bgH Poly(A) region were synthesised (Twist Bioscience). Pause-NfKB-MiniP-RedFirefly-PEST-bgHpoly(A) construct was also synthesised (Twist Bioscience). Each TRE (Supplementary Table 1) was subcloned from commercially available pNL[TRE-NLuc-PEST-Hygro] constructs (Promega, UK) using *Nhe*I and *Hind*III into their respective synthesised DNA vectors (Twist Bioscience, Supplementary Table 1). From here, primers (Supplementary Table 2) were used to amplify the 1X sequence whilst introducing an additional restriction site at the 3’ end (*BamH*I for all apart from CRE and NfkB, which were *Hind*III). The PCR product was digested immediately with *BamH*I and *Bgl*II (FastDigest, 30 min digest, 37 °C) before PCR cleanup. In parallel, the vector was digested with *Bgl*II (NFAT, SRE and SRF-RE) or *Hind*III (CRE and NfkB) in the presence of calf intestinal phosphatase (CIP; New England BioLabs). PCR products were then combined with 50 ng of digested vector, 1U or *Bgl*II, T4 Ligase and T4 ligase buffer before thermocycling (as described in Fig. 2A). Thermocycler cycled between optimal digest temperatures (37 °C for 4 min) and ligase temperatures (16 °C for 10 min) 25 times to produce concatenate sequences (Fig. 2A). After cycling, an additional 15 min at 16 °C was added to enhance ligation before denaturing the enzymes (10 min at 85 °C). Products were transformed, and colony PCR was performed to identify concatenates based on a stepwise increase in base-pairs. Positive results were confirmed by Sanger sequencing (Source-Bioscience, UK). The CRISPR/Cas9 homology directed repair template constructs, pAAV_hΒ2M_KI_HDRT_FLAG-CD52GPI-PolyA_CRE2X_NLuc_Rev_KI_CO (pCRE2X-NLuc-PEST-KI) was ordered from GenScript (Oxford, UK). The CRE2X region of the plasmid was excised by *Nhe*I and *Hind*III digestion at 37 °C for 30 min (FastDigest, Thermo Fisher). The same digestion was performed on pNFAT1X-NLuc-PEST, pNFAT 2X- pNFAT2X-NLuc-PEST, pNFAT3X-NLuc-PEST and pNFAT4X-NLuc-PEST plasmid DNA to excise the NFAT binding regions. The NFAT concatenate sequences were then subcloned into the digested pCRE2X-NLuc-PEST-KI to yield pNFAT1X-NLuc-PEST-KI, pNFAT2X-NLuc-PEST-KI, pNFAT3X-NLuc-PEST-KI and pNFAT4X-NLuc-PEST-KI. Concatenate NFAT constructs were digested for 15 minutes (37 °C) with *Nhe*I and *Hind*III (FastDigest, ThermoFisher Scientific). Samples were loaded into a 1% agarose (w/v) 1X TAE gel and run at 120 V for 30 min before visualisation on an iBright visualiser (Thermo Fisher Scientific).

### General cell culture and transfection

All adherent cell lines (HEK293T; ATCC, HeLa; ATCC, HEK293; ATCC, A549; gift from AstraZenca, UK) were grown (37 °C, 5% CO_2_) in Dulbecco’s modified Eagle’s medium (DMEM)/F12 (1:1) with GlutaMAX™ (Thermo Fisher, UK), supplemented with 10% (v/v) foetal bovine serum (FBS, Sigma, UK), and 1% antibiotic-antimycotic (Sigma, UK). Cells were grown up to 85–90% confluency in T75-cm^2^ flasks before passaging. All cell types are routinely tested for mycoplasma contamination every 3 months. Unless otherwise stated, all transfections were performed in six-well (Greiner, Bioscience, UK) plates, at 75–80% confluency with a total DNA mass of 2 μg per well. Transfections were performed using 1:2 (w:v) DNA: lipofectamine 2000 (Thermo Fisher Scientific; HEK293T and HeLa) or 1:3 (w:v) DNA:Transit-LT (Mirus; HEK293 and A549) as per the manufacturer’s instructions. All transfections were performed for 24 h before reseeding. For A549 cell transfections, total DNA of 5 ug was used, and cells were transfected in a T25 cm^2^ (Greiner-One, UK) flask at 80% confluency. For HEK293T experiments, receptor DNA mass for each transfection was optimised per receptor (Supplementary Table 3).

### Cell seeding and nano-luciferase reporter assay

#### General

All cells, regardless of cell type, were trypsinised and resuspended in DMEM/F12 (1:1) with GlutaMAX™ but without phenol red (ThermoFisher Scientific, UK) and diluted to a density of 50,000 cells per 100 μL. To generate serum-starving conditions, the assay media was supplemented with 0.5% (v/v) foetal bovine serum (FBS, Sigma, UK). Although low, the FBS percentage is not reduced to 0 for starvation conditions, in order to facilitate cell adherence. However, it must be noted that residual serum may contribute to basal signalling, particularly for SRE and SRF-RE reporters, requiring consideration when interpreting their absolute basal luminescence production. Then, 100 μl of diluted cells were seeded onto poly-L-lysine-coated (except A549 and HeLa) white 96-well plates (Greiner- Bioscience, UK). A549 and HeLa cells were seeded onto human fibronectin (45-min coat; Merck, UK) coated 96-well plates (Greiner-Bioscience, UK). The next day (16–20 h later), 100 μl of each ligand and concentration, diluted to a 2X concentration in DMEM/F12 without phenol red and 0.2% bovine serum albumin (BSA; such that a 1:1 dilution results in 1X final concentration). Vehicle-treated cells were stimulated with the solvent its respective ligand was diluted in—either dH_2_O, DMSO, or Ethanol. Cells were only seeded from row B-G and column 2–11, with a 100 μl PBS border, to reduce evaporation plate-effects. Moreover, ordering of reporter construct on plates was alternated to confirm no plate-effect of construct positioning. No condition was ever alone on a plate; the fourth condition would be seeded with additional NFAT 1X to verify no plate-plate variability. Cells were stimulated in duplicate for all conditions, and kept in growth conditions (37 °C, 5% CO_2_) for 3 h unless stated otherwise. To investigate G_q/11_ contributions to NFAT responses, cells were pretreated for 30 min (experiments were performed in parallel with non-YM, which had sham media changes) with 100 nM YM254890, an established G_q/11_ inhibitor^38^. An equal concentration of YM254890 was also added to the ligand assay buffer to maintain concentration after ligand addition. When PTX, (Gibco) was used to investigate G_i/o_ contributions, cells were pretreated with 200 ng/ml PTX for 16 h before ligand addition, and PTX concentration was maintained throughout stimulation. To inhibit the action of small G protein RhoA, cells were pretreated for 30-min with 30 μM Rhosin hydrochloride (Tocris Bioscience, Wiltshire)^78^. With all inhibitor experiments, cells were subject to the same concentration of each inhibitor for the entire experiment, and solvent concentrations were matched across treatments. Non-treated cells were always media-changed (sham) when inhibitors were added. To inhibit the action of IP_3_, HEK293T cells were co-transfected with NFAT 3X-NLuc-PEST and an IP_3_ kinase C construct, to constitutively phosphorylate IP_3_, preventing it from activating IP_3_Rs^59^.

### Ligand procurement and dilution

Adenosine, isoprenaline, oxytocin and Sphingosine-1-Phosphate (10 mM in DMSO) were purchased from Sigma-Aldrich (Merck, Dorset, UK). Adrenaline, carbachol, forskolin, UTP, histamine, Hu210, and dopamine were purchased from Tocris Bioscience (Wiltshire, UK). Parathyroid hormone (PTH(1–34)), GLP-1(7–36) and GIP(1–42) was purchased from Bachem (Bubendorf, Switzerland). All peptides were diluted to 1 mM in dH_2_O containing 0.1 BSA and aliquoted before storing at −20 °C. Hu210 was received prediluted in 100% ethanol to a stock concentration of 10 mM. Adenosine, adrenaline, histamine, UTP and dopamine were dissolved to a stock concentration of 10 mM in sterile dH_2_O and aliquots stored at −20 °C. Carbachol was dissolved to a stock concentration of 1 mM sterile dH_2_0 and aliquots were stored at −20 °C. Forskolin was dissolved in tissue culture grade DMSO to a stock concentration of 10 mM and stored in and aliquots stored at −20 °C. DMSO was purchased from Sigma-Aldrich (Merck, Dorset, UK) and aliquoted to prevent water absorption. Pre-dissolved Sphingosine-1-Phosphate (S1P; 10 mM in DMSO) was purchased from Sigma-Aldrich (Merck, Dorset, UK). Where appropriate and on the day of assay, dissolved stocks were serially diluted in a sterile environment and in their specific solvent.

### Reporter assay luciferase detection

Nano-luciferase (NFAT) assays were performed for 10 min before the stimulation period ended, plates were removed from the incubator and left to equilibrate to room temperature (RT). Then, stimulation media was aspirated from all cell-containing wells using a multi-channel pipette. This step was repeated to ensure the removal of all media. Cells were lysed with 50 μl of 1X passive lysis buffer (PLB Promega, UK), supplemented 1:1000 with Nano-Glo® substrate (furimazine; Promega, UK). This process was performed by individual technical repeat (by row), with a multi-channel. Cells were shaken in the dark for 15 min before luciferase activity measurement.

For the CRE and NfkB assay, plates and cells are treated identically until PLB addition. Non-supplemented 1X PLB (40 μL) was added to aspirated wells, and cells were shaken for 15 min to induce lysis. Then 10 μl of LARII buffer (Dual-Luciferase® Reporter System, Promega, UK), which contains D-luciferin (Firefly and red-firefly substrate), was added to wells, one technical repeat at a time using a multi-channel. Plates were then shaken for a further 3 min before luciferase activity measurement.

For SRE and SRF-RE assays, plates and cells are treated identically until PLB addition. 1X PLB supplemented with 10 μM coelenterazine-h (Nanolight Technology, USA) was added to aspirated cell-containing wells (50 μl). Cells were then shaken in the dark for 15 min before luciferase activity measurement.

### Luciferase activity measurement

Unfiltered luminescence measurements were read using a CLARIOstar Plus® (BMG Labtech, UK) after a 15-min lysis and luminescent substrate incubation. The 15-min incubation was performed to provide uniform cell lysis across all wells and ensure luciferases were in the slow and linear decay phase of luminescence output rather than non-linear rapid decay, which could maximise luciferase addition timing-induced error. All measurements were performed with a 1-s interval reading time (exposure time). Apart from A549 measurements, all measurements were taken using the Enhanced Dynamic Range (EDR) gain setting. Background luminescence in empty wells was negligible (10 s of RLU compared to ~10^4.5^–10^7^) so was not deducted. For A549 measurements, 4095 gain was used to maximise signal intensity.

### HDRT generation and purification

The following process was performed with all the following constructs: pNFAT 1X-NLuc-PEST-KI, pNFAT 2X-NLuc-PEST-KI, pNFAT 3X-NLuc-PEST-KI and pNFAT 4X-NLuc-PEST-KI. The process was first performed on the pCRE2X-NLuc-PEST-KI to confirm our editing strategy could produce reporter T cells (Supplementary Fig. 4). Gradient polymerase chain reaction (PCR) was performed with T7 forward (TAATACGACTCACTATAGGGCGA) and SP6 reverse (GTATTCTATAGTGTCACCTAAA) primers (0.5 µM of each primer, per 1 ng of template DNA) to ascertain optimal annealing temperatures between 55 and 65 °C. PCR was always performed with PhusionPlus MasterMix (Thermo Scientific, F-631) with the same cycle conditions (Supplementary Table 4). After visualisation of yield a 1% agarose (in TAE) gel (100–200 V depending on tank size) using SYBR safe (Invitrogen), 55.8 °C was the optimal annealing temperature selected for all templates. The HDRT from each KI construct was amplified (55.8 °C anneal; Supplementary Table 4) and the DNA purified using AMPure XP beads (Beckman Coulter; 1:0.6, v:v, PCR:beads) and Pierce™ Protein Concentrators PES, 100 K MWCO filtration columns (Thermo Scientific, 88532). Purified DNA was diluted to 1.6–2 µg/µl in RNAse-free water before aliquoting and storage (−20 °C).

### Primary human T cell culture, genome editing and flow cytometry

CD8^+^ T cells were isolated from a healthy donor Leukopak (BioIVT), as per Human Tissue Act (TCA) regulations ^79^. Ficoll centrifugation with SepMate tubes (StemCell) was used to isolate peripheral blood mononuclear cells (PBMCs) from blood. Negative selection kits (StemCell) were then used to isolate CD8^+^ T cells before aliquoting and storage (−140 °C). Vials from a single donor were thawed and CD8^+^ T cells were grown in AIM-V Medium (Thermo Fisher; CAT# 12055091) media (Gibco, Thermo Fisher, Paisley, Scotland) supplemented with 5% human serum (Merck, CAT# H4522) and 300 IU/ml IL-2 (BioLegend, 589108) at 37 °C with 5% CO_2_. Cells were activated using ImmunoCult Human CD3/28 T cell activator (StemCell, 10971) as per the manufacturer’s instructions. Three days later, cells were pretreated for an hour with 1 µM DNAPKi (AstraZeneca, UK) before pelleting (300×*g* for 5 min) and washing with 1X PBS. Cells were resuspended to 1 × 10^5^ cells/µl in Lonza resuspension buffer P3. Cells were then combined with RNP and HDRT DNA (2 µg/1 × 10^6^ cells) before electroporation in a Nucleofector™ Cuvette (1 × 10^6^ cells/100 µl/cuvette), in a Lonza 4D (pulse code: EH-100). Per 1 × 10^6^ cells, RNPs were made up of 125 pmol single guide RNA (sgRNA; GAGTAGCGCGAGCACAGCTA, Merck) for the    (β2M gene and 50 pmol Cas9 (AstraZeneca, UK), incubated for 30 min at RT. For KI reactions, electroporated cells were transferred into pre-prepared 96 deep well plates (Thermo Scientific, AB-0661), with prewarmed recovery media containing 1 µM DNAPKi (no DNAPKi for Mock or KO reactions). Cells were cultured at 37 °C, 5% CO_2_ for 2 h before Benzonase addition. Media was replaced 24 h post electroporation, removing benzonase and DNAPKi. Cells were cultured at 37 °C, 5% CO_2_ and the medium was refreshed when required (as determined by media colour change).

As the KI constructs would deliver a FLAG-CD52-GPI gene downstream of the β2M promoter in the 5’ to 3’ direction, we could assess KI-efficiency by staining for FLAG, and KO efficiency by staining for Β2M. Before assaying, a 1 × 10^5^ cell samples from each condition were assessed via flow cytometry. Concentrated and washed cells were stained with Zombie Violet™ Fixable Viability Kit (BioLegend, 423114, 1 in 500 1X PBS) for 25 min at RT, APC anti-Β2M Ab (BioLegend, 316312, 1 in 100 FACS buffer) and PE anti-FLAG Ab (BioLegend, 637310, 1 in 100 FACS buffer). Both antibody stains were performed for 30 min at 4 °C. Cell viability, FLAG-CD52 expression, and Β2M expression were assessed using the BD LSR-Fortessa Cell Analyzer (BD Bioscience).

### Primary human T cell CRE-NLuc-PEST reporter assay setup

Edited cells were grown and split to maintain populations between 2–3 × 10^6^ cells per ml in 24-well plates (Costar®). Cells were counted (Vi-Cell) and viable cell counts used to dilute cells in growth media to a density of 1 × 10^6^ cells per ml, before seeding 1 ml into wells of a 24-well plate. Cell populations were stimulated with Adenosine, Adrenaline, Forskolin and Dopamine at varying final concentrations. Populations of DMSO ligand vehicle (0.1% v/v DMSO) and dH_2_O vehicle (1% v/v dH_2_O) treated cells were also assayed to act as no ligand controls, for baseline correction. After ligand addition, cells were stimulated for 4 h (37 °C with 5% CO_2_) before resuspension and transfer of 100 μl cell suspension, in duplicate, to a white 96-well plate (Costar®).

### Primary human T cell NFAT-NLuc-PEST reporter assay setup

Edited cells were grown and split to maintain populations between 2–3 × 10^6^ cells per ml in 24-well plates (Costar®). Half of the media from each well (600 μL) was replaced with IL-2-free media 18 h before assay start, then again repeated 4 h before assay start, leading to a 1:4 dilution of IL-2. Cell viability counts (Vi-cell) were used to scale seeding densities of the four populations of cells (NFAT 1X-KI, NFAT 2X-KI, NFAT 3X-KI and NFAT 4X-KI) such that the density of FLAG-CD52+ cells was 290,000 FLAG-CD52^+^ cells per ml (Supplementary Table 5). Wells containing 500 µl (145,000 FLAG-CD52^+^ cells) of diluted cells were stimulated with either 0.1% DMSO (v/v, Sigma (D2650-100 ml) or 10 μM Sphingosine-1-Phosphate (S1P; in DMSO). After 4 h stimulation (37 °C with 5% CO_2_), cells were resuspended before transfer of 100 μl cell suspension, in duplicate, to a white 96-well plate (Costar®).

### Luciferase activity measurement of T cells

Luciferase activity was detected using the Promega Nano-Glo® Luciferase Assay System (Promega, UK, N1120), as per the manufacturer's protocol. In brief, Nano-Glo® substrate (furimazine) was diluted 1:50 into Nano-Glo® buffer, before being added 1:1 (v:v) to cell suspensions. Plates were shaken for 15 min to allow for cell lysis before luminescence detection in an Envision 2104 Multiplate Reader, PerkinElmer.

### Data analysis

#### Reporter assays

Data analysis was performed in GraphPad Prism 10 (GraphPad Software, San Diego, CA, USA). Raw relative light unit data is displayed as the mean ± standard error of the mean (SEM) for at least three independent experiments performed with two to four technical repeats, unless stated otherwise. SEM calculation was performed per individual experiment, not per technical repeat. Mean ± SEM fold induction was generated by dividing all results by the vehicle-treated mean. Both raw and fold induction datapoints were fitted with a three-parameter logistic regression to obtain log[EC50]’s and FI Emax (GraphPad Software).

#### Statistics and reproducibility

To assess the statistical significance of differences, we performed paired one-way ANOVAs with Fisher’s LSD for specific comparisons. First, normality was assessed using quantile-quantile plots before performing ANOVAs with Fisher’s LSD when significance was present. If the original ANOVA returned no significant difference, ‘ns’ was added to the log[EC50]/FI Emax plot. The Fisher’s LSD^80^ was chosen for multiple comparisons as we are making distinct planned comparisons (NFAT 1X vs NFAT 2X, NFAT 1X vs NFAT 3X and NFAT 1X vs NFAT 4X) between NFAT 1X and each concatenated reporter. The threshold for significance was set at *p* < 0.05. Significance was then displayed by a coloured asterisk next to the data, which was statistically different to NFAT 1X as per Fisher’s LSD. Alongside all stated *p* values are the mean differences with 95% confidence intervals (CIs) such that *p* = X (mean difference (MD) = Y ± SEM [lower, upper 95% CI]). All statistical tests were performed in GraphPad Prism 10. All sample sizes were determined, and datasets analysed, in accordance with the guidelines described by ref. ^81^. All experiments were appropriately controlled using ‘system pathway’ agonists. If any of these pathway controls generated inappropriate responses, then the entire dataset was removed from analysis if not shown to be experimental error.

### Ethics statement

All human biological samples were commercially acquired (BioIVT, UK). AstraZeneca ensures that commercial sources have appropriate patient consent and ethical approval for the collection of research samples, including use by for-profit companies. The AstraZeneca BioBank in the UK is licensed by the Human Tissue Authority (Licence No. 12109) and has National Research Ethics Service Committee (NREC) approval as a Research Tissue Bank (RTB) (REC No. 22/ NW/0102), which covers the use of samples here.

### Reporting summary

Further information on research design is available in the Nature Portfolio Reporting Summary linked to this article.

## Supplementary information

Supplementary Information

Description of Additional Supplementary Files

Supplementary Data 1

Supplementary Data 2

Supplementary Data 3

Supplementary Data 4

Supplementary Data 5

Supplementary Data 6

Reporting Summary

## Data Availability

Source datasets for the graphs and charts in the main figures are given as Supplementary Data Files 1–6. Any remaining information, including the primary data for all pharmacological investigations are available from the corresponding authors on reasonable request. DNA sequencing files, etc. will be made available via (https://www.openresearch.cam.ac.uk/data/deposit).
