# Supplementary material for: Enhancement of a nuclear factor of activated T cells (NFAT) reporter for the study of G protein-coupled receptors
Source: Commun Biol. 2026 Apr 26;9:882. doi: 10.1038/s42003-026-10110-5 (PMC13323705; doi:10.1038/s42003-026-10110-5)
Supplement: Supplementary file 1 — Supplementary Information [file 42003_2026_10110_MOESM1_ESM.pdf]

Supplementary information

Enhancement of a Nuclear Factor of Activated T cells (NFAT) Reporter for the Study of G Protein-Coupled Receptors.

Edward Wills[1], Anjana Saji[1], Jonathan Sumner[2], Aman Khan[1], Claudia M. Sisk[1], Mona Shehata[3], Benjamin Taylor[2], Graham Ladds[1*]

1 Department of Pharmacology, University of Cambridge, Tennis Court Road, Cambridge, CB2 1PD, UK.

2 Cell Immunology, Discovery Sciences, R&D, AstraZeneca, Cambridge, UK

3 Bioassay, Biosafety and Impurities, BioPharmaceutical Development, AstraZeneca, Cambridge, UK.

* To whom correspondence may be addressed

Content

9 Supplementary Figures

5 Supplementary Tables

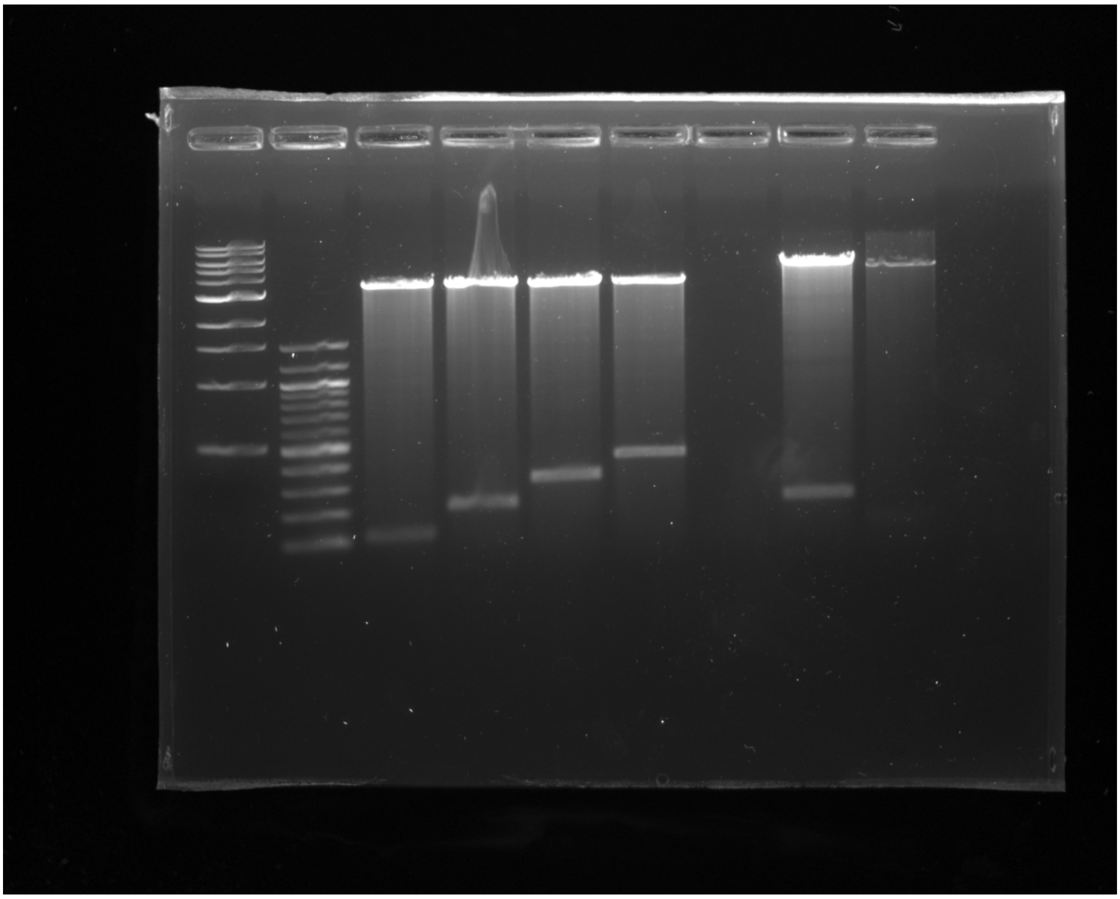

Supplementary Figure 1. Uncropped gel displaying restriction digestion of concatenated NFAT-RE triplicate binding sites. Visualisation of three concatenate versions of NFAT-RE; NFAT 2X (211 bp), NFAT 3X (307 bp), NFAT 4X (403 bp), visualised on a 1% agarose w/v TAE gel, ran for 30 minutes at 120 V.

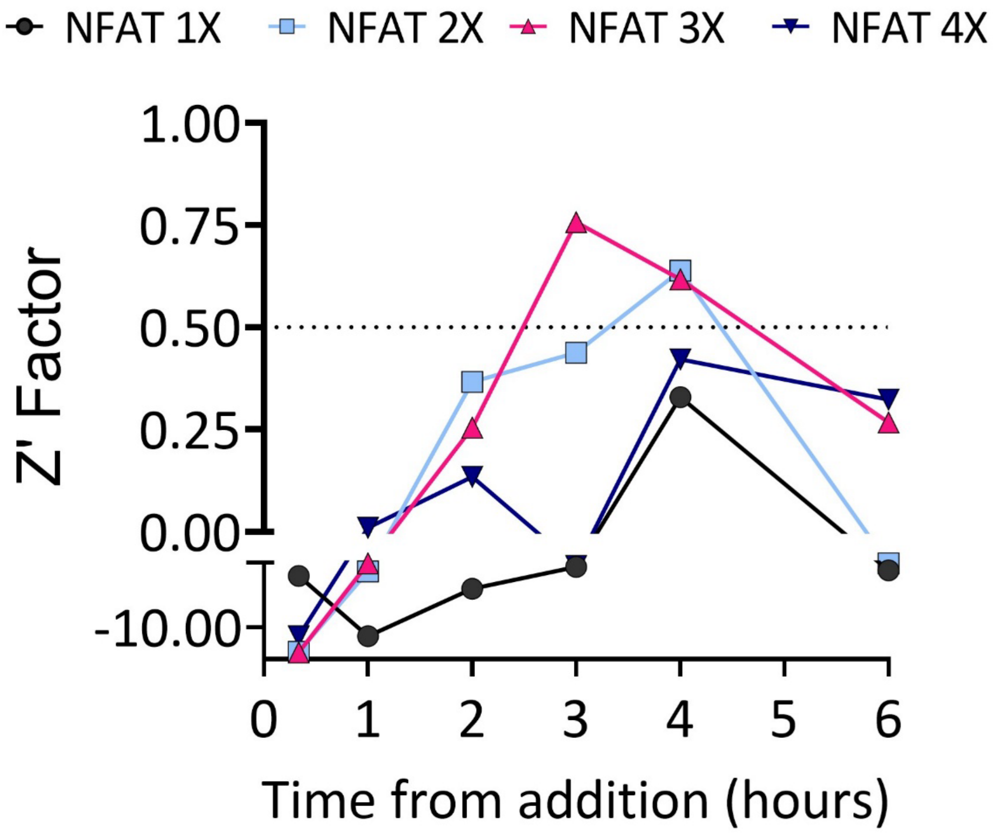

Supplementary Figure 2. NFAT 2X and NFAT 3X reporter constructs produce highest Z' scores between 2 and 4 hours of stimulation. Z scores for each reporter (NFAT 1X, NFAT 2X, NFAT 3X and NFAT 4X) were calculated by comparing vehicle and positive control stimulated responses across 6 time-points.

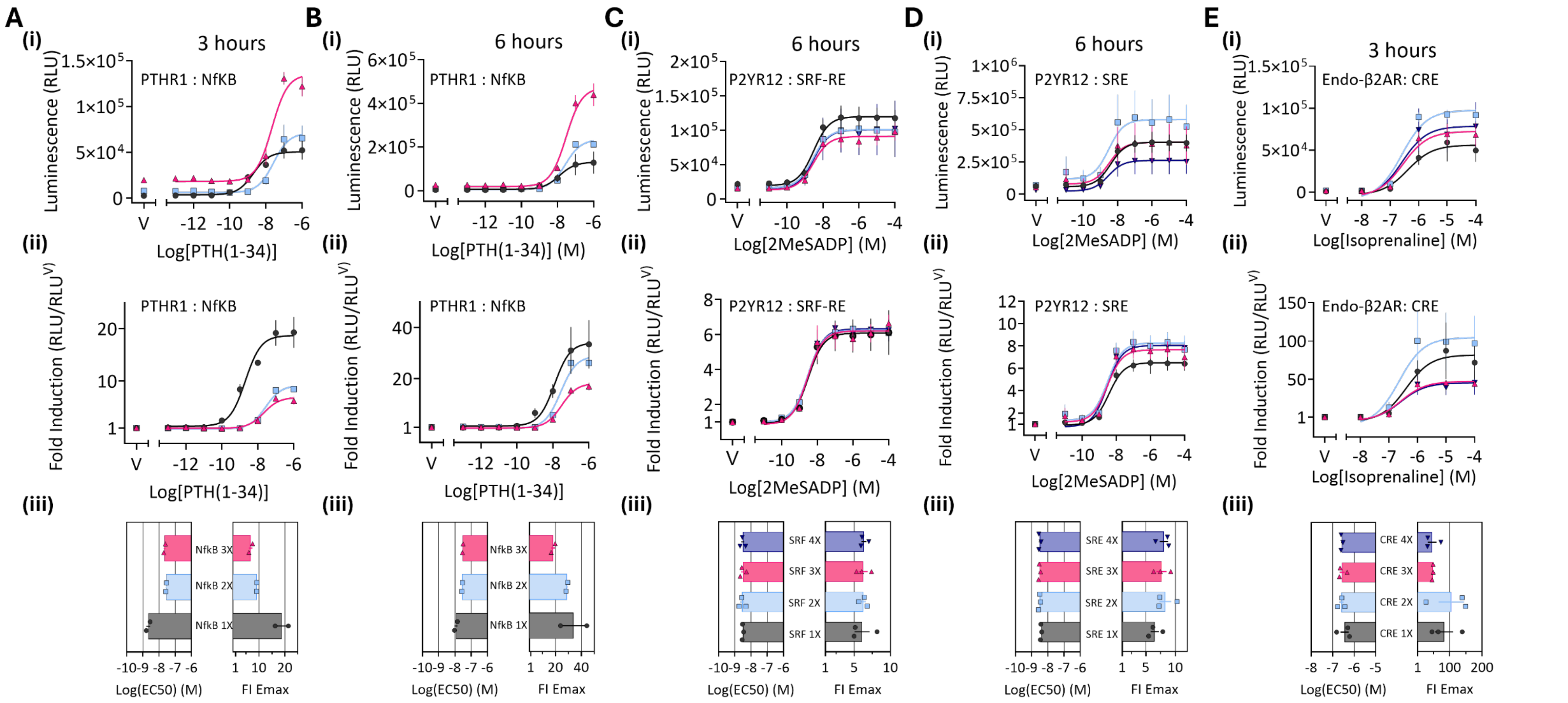

Supplementary Figure 3. Concatenation of other GPCR sensitive GREs decreases or does not affect responsivity in HEK293T cells. Mean ± SEM (*n* =2-3) luminescence responses, in relative light units (RLU) to increasing concentrations of GPCR agonist (**A-E(i)**). For all types of concatenate reporters, the original 1X construct is denoted with grey circles, 2X with blue squares, 3X with pink upward triangles and 4X with purple downward triangles. To assess responsivity, Mean ± SEM Fold induction (FI) responses are generated by dividing each RLU value by the vehicle (RLU/RLU^{Vehicle} (**A-E(ii)**). All dose responses are fitted with three-parameter non-linear regression and Log(EC50) (M) and Fold Induction mean ± SEM maximal responses (FI Emax) compared (**A-E(iii)**) HEK293T cells were transfected with PTHR1:pcDNA3.1(+) with 1X, 2X and, 3X concatenate Nuclear Factor of Kappa Beta (NfkB) – Red Firefly Luciferase (RedF) reporter (NfkB-RedF-PEST) and before stimulation with PTH(1-34) for 3 (**A**) and 6 (**B**) hours (n = 2). P2YR12-HEK293T stable cells were transfected with 1X, 2X, 3X, 4X concatenates of the quintuplet Serum Response Factor Response Element (SRF-RE) - Green *Renilla* Luciferase (GrRLuc) reporter (SRF-RE-GrRLuc-PEST; n = 3 **C**) and Serum Response Element (SRE) Renilla Luciferase (RLuc) reporter (SRE-Rluc-hPEST, n =3 ;**D**). Transfected cells were stimulated with 2MeSADP for 6 hours (**C-D**). The endogenous Beta 2 Adrenoceptor (β2AR) expressed in HEK293T cells was stimulated with Isoprenaline after transfection with 1X, 2X, 3X and 4X duplex cAMP Response Element (CRE) – Firefly Luciferase (FLuc) reporters (CRE-FLuc-PEST, n = 3; **E**).

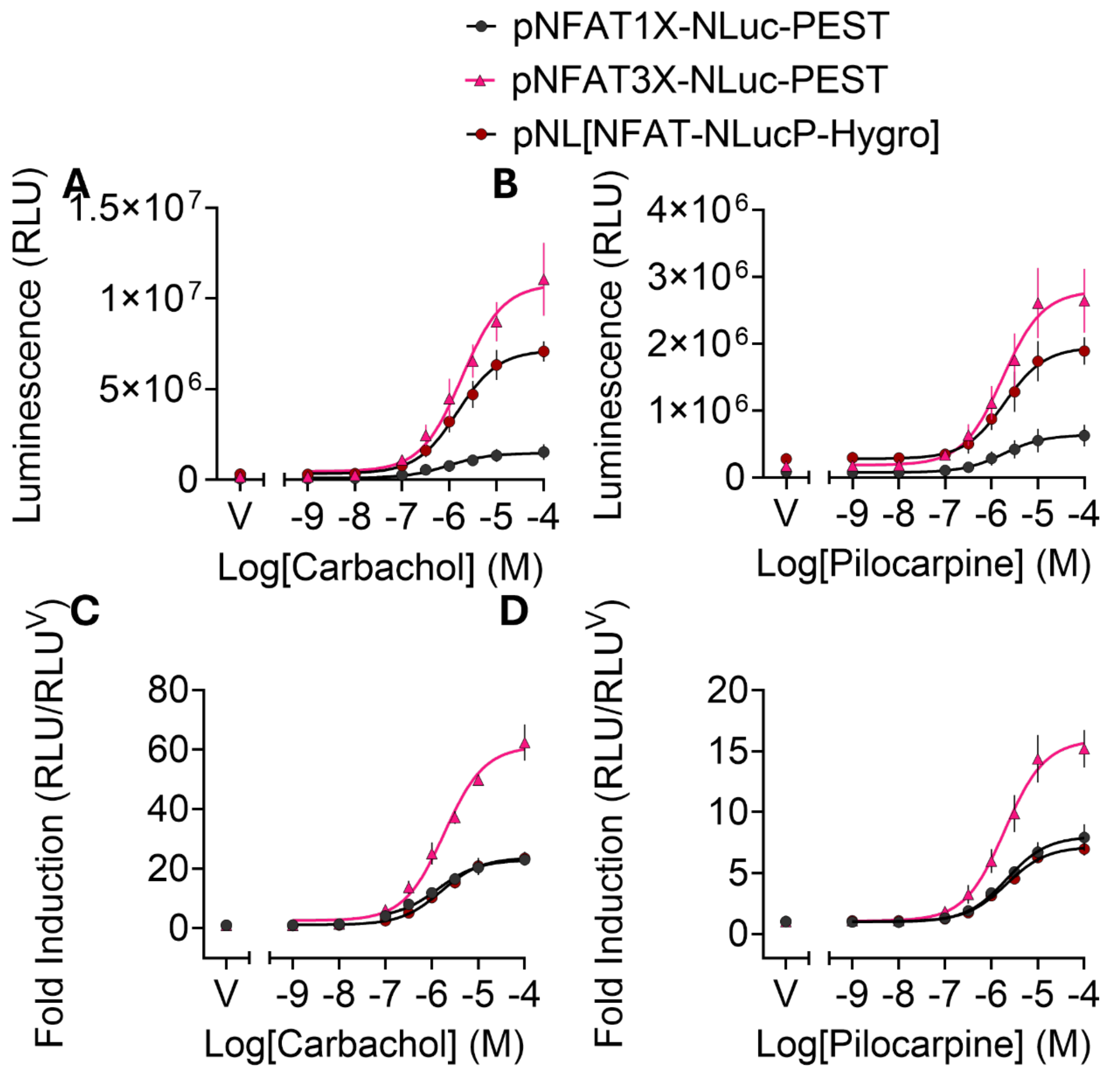

Supplementary Figure 4. The NFAT 3X reporter provides largest response range to M_3R signalling when benchmarked in HEK293T cells. (**A-B**) Mean ± SEM (*n* = 3) luminescence responses, from HEK293Ts transfected with classically $G\alpha_q$-coupled M_3R with pcDNA3.1(+) and either pNFAT1X-NLuc-PEST (**grey circles**), pNFAT3X-NLuc-PEST (**pink upward triangles**), and pNL[NFAT-NLucP-Hygro] (**red circles**). Measured in relative light units (RLU) to increasing concentrations of full agonist carbachol (**A**) and partial agonist pilocarpine (**B**). To compare responsivity, mean ± SEM fold induction (FI) responses were generated by dividing each RLU value by the vehicle (RLU/RLU^{Vehicle} (**C-D**). Differences in vehicle treated luminescence are displayed as insets in (**A**) and (**B**).

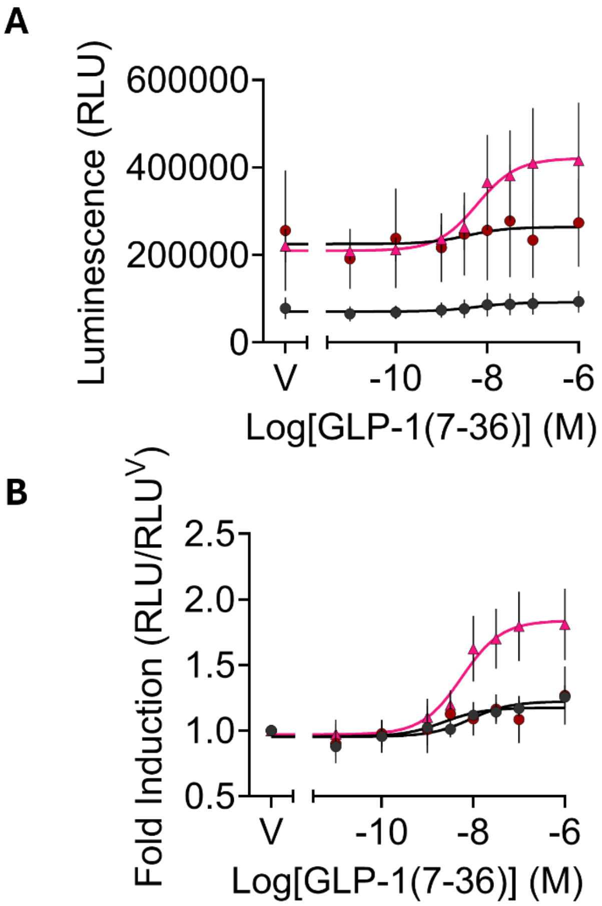

Supplementary Figure 5. The NFAT 3X reporter provides largest response range to GLP-1R signalling when benchmarked in HEK293T cells. (**A-B**) Mean ± SEM (*n* = 3) luminescence responses, from HEK293Ts transfected with classically Gα_s-coupled GLP-1R with pcDNA3.1(+) and either pNFAT1X-NLuc-PEST (**grey circles**), pNFAT3X-NLuc-PEST (**pink upward triangles**), and pNL[NFAT-NLucP-Hygro] (**red circles**). Measured in relative light units (RLU) to increasing concentrations of agonist GLP-1(7-36) (**A**). To compare responsivity, mean ± SEM fold induction (FI) responses were generated by dividing each RLU value by the vehicle ($RLU/RLU^{Vehicle}$) (**B**).

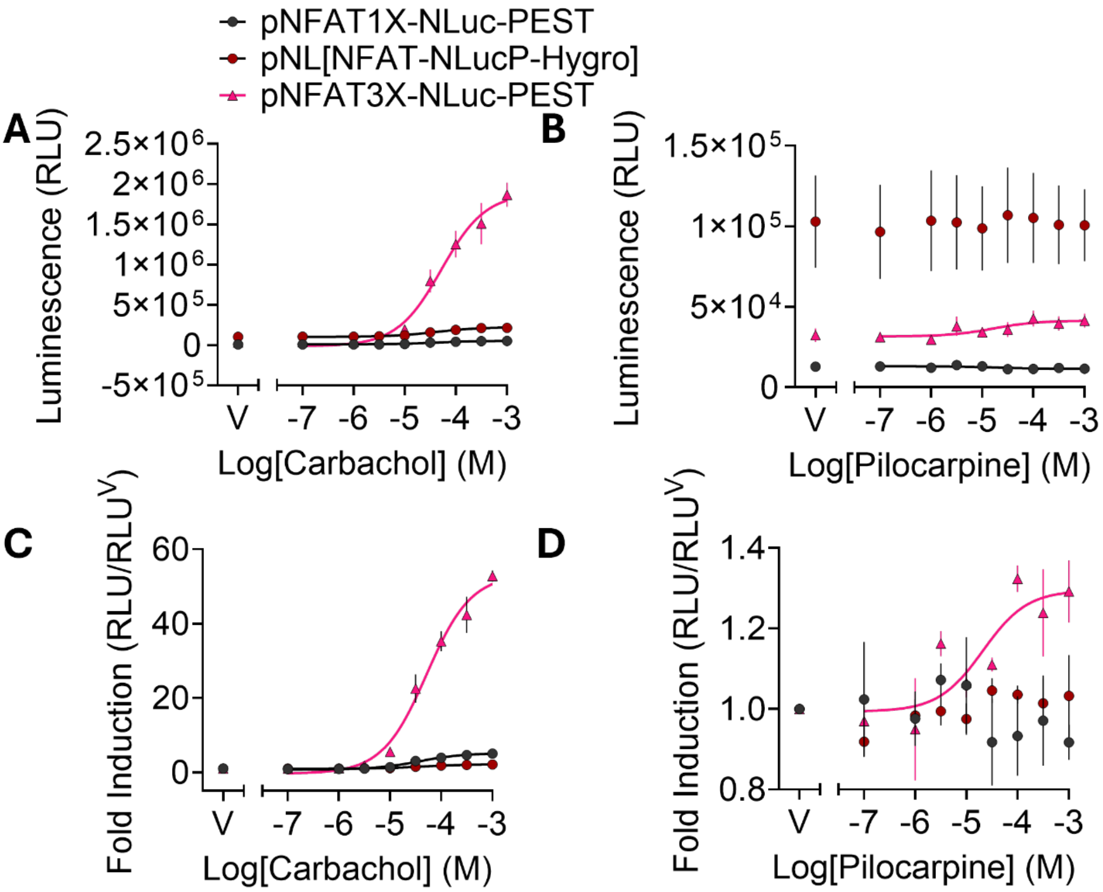

Supplementary Figure 6. The NFAT 3X reporter provides largest response range to endogenous M$_3$R signalling when benchmarked in HEK293T cells. (**A-B**) Mean ± SEM (*n* = 3) luminescence responses, from HEK293 WTs transfected with classically with pcDNA3.1(+) and either pNFAT1X-NLuc-PEST (**grey circles**), pNFAT3X-NLuc-PEST (**pink upward triangles**), and pNL[NFAT-NLucP-Hygro] (**red circles**). Measured in relative light units (RLU) to increasing concentrations of full agonist carbachol (**A**) and partial agonist pilocarpine (**B**). To compare responsivity, mean ± SEM fold induction (FI) responses were generated by dividing each RLU value by the vehicle (RLU/RLU^{Vehicle} (**C-D**). Differences in vehicle treated luminescence are displayed as insets in (**A**) and (**B**).

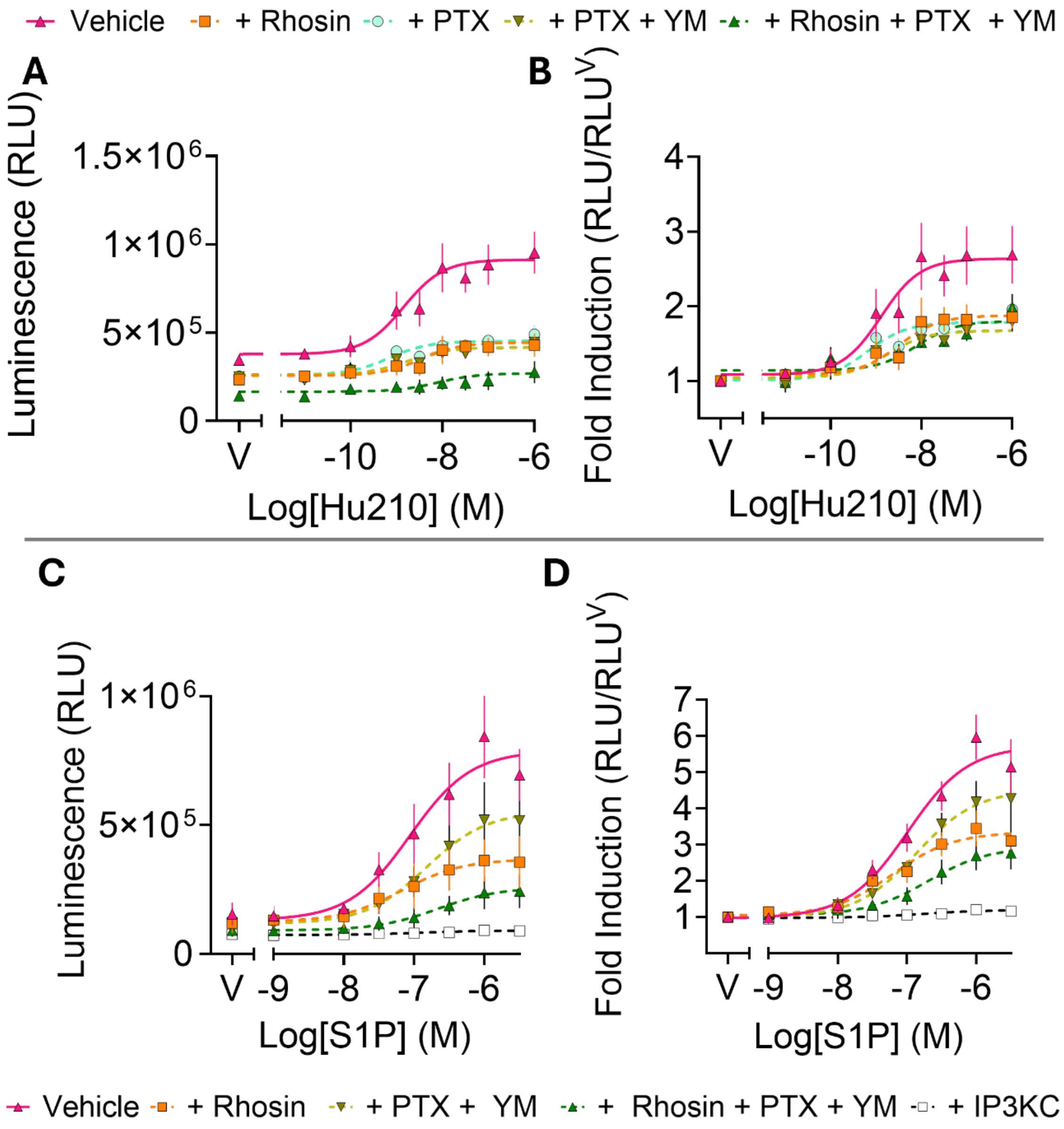

Supplementary Figure 7. G$\alpha_{i/o}$- linked GPCR mediated NFAT signalling in HEK293T cells is partially inhibited by G$\alpha_{i/o}$, G$\alpha_{q/11}$, and RhoA blockade. Mean ± SEM (*n* = 4) luminescence responses, from HEK293T's transfected with NFAT 3X and CB_1R (**A-B**) or empty vector (pcDNA3.1) (**C-D**) before stimulation with Hu210 and S1P, respectively. (**A**) Cells were stimulated in the presence of, and after 16 hour PTX treatment, after 30 minute pretreatment of 100 nM $G_{q/11}$ blocker YM254890 (YM), and 30-minute pretreatment of 30 µM Rhosin hydrochloride. Different groups were treated with the described combinations of inhibitors for the duration of the assay. Relative luminescence units (RLU; **A and C**) are plotted and fold induction (FI) responses (**B and D**) generated by dividing each RLU value by the vehicle ($RLU/RLU^{Vehicle}$). All dose responses are fitted with three-parameter non-linear regression fits. With S1P treated cells, one subset of HEK293T cells were transfected with IP3KC (**C-D**) to constitutively convert IP_3 to IP_4, thus eliminating IP_3 signalling.

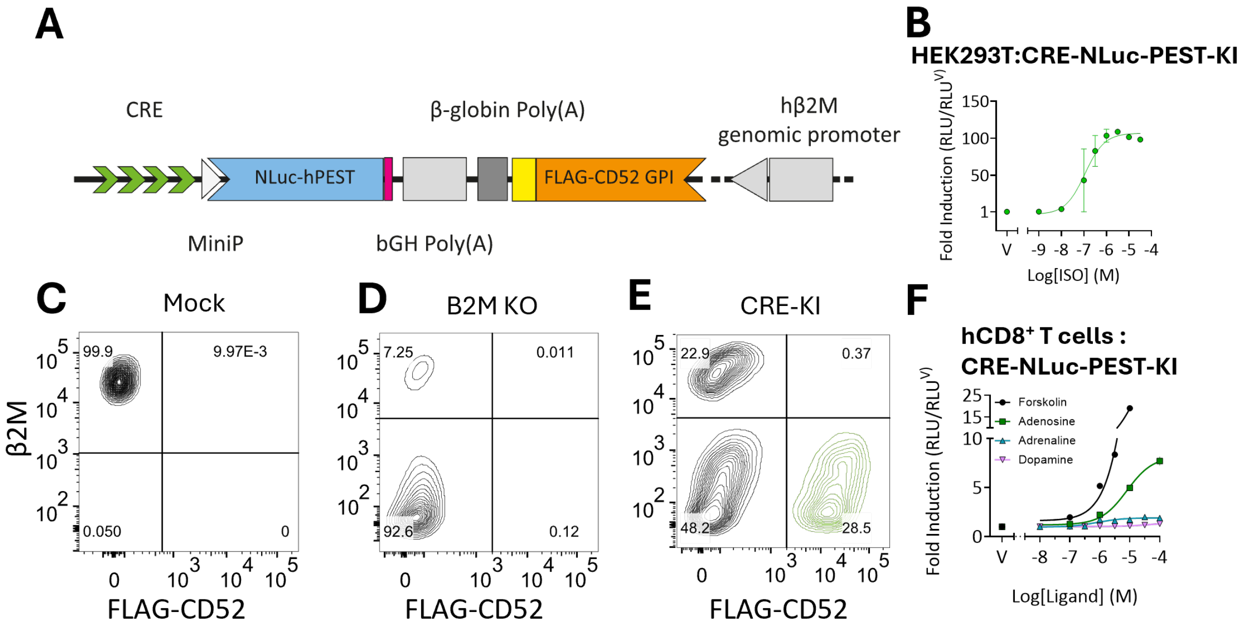

Supplementary Figure 8. Development and validation of pilot CRE-NLuc-PEST human CD8+ T cell reporter cells via CRISPR knock-in. (**A**) CRE homology directed repair template (HDRT) was designed to incorporate the reporter and FLAG-tagged, GPI-Anchored, CD52 gene expressed off the endogenous Beta-2-Microglobulin promotor (B2M). (**B**) Mean ± SEM (n = 2) fold induction (FI) from vehicle ($RLU/RLU^{Vehicle}$) responses of HEK293T cells transfected with pCRE-NLuc-PEST-KI and stimulated with isoprenaline (ISO) to activate the endogenously expresses β-2 adrenoceptor. (**C-E**) Editing efficiency of CRE knock in (CRE-KI) $CD8^+$ T cell population is determined via flow-cytometry of anti-B2M and anti-FLAG-stained cells. Contour plots of mock-edited (**C**)**,** B2M knock out (KO; 92.6 %) (**D**), and CRE-KI (28.5 %) (**E**) are shown, where high FLAG-CD52 (+) and low B2M (-) populations of cells are considered successful for CRE-KI knock in (green). Cells are then left to recover for 2-weeks before functional validation. (**F**) Mean ± SEM FI responses (single donor, performed in duplicate) from populations of CRE-NLuc-PEST edited $CD8^+$ T cells, to adenylate cyclase activator forskolin (**black circles**) and G_s-coupled GPCR agonists adenosine (**green squares**), adrenaline (**blue upward triangles**), and dopamine (**pink downward triangles**).

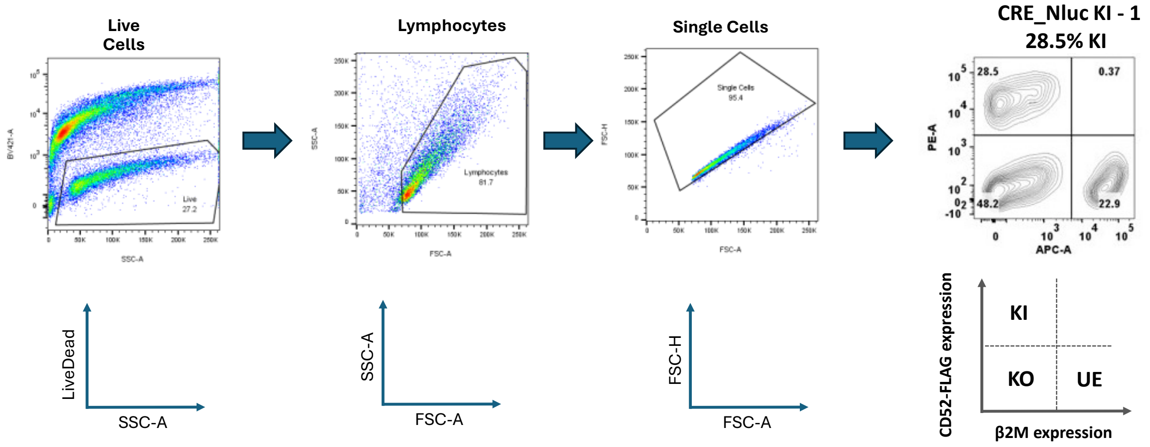

Supplementary Figure 9. Gating strategy for CRISPR Knock-in validation. (1) Live cell gate (2) Lymphocyte gate (3) Doublet exclusion gate (4) % FLAG-CD-52+/ B2M- (B2M/FLAG-CD52+ KI cells). 1.5 x 10^5 cells were pelleted (300 x g for 5 min). Cells were washed once in PBS and stained with Zombie Violet™ Fixable Viability Kit (Biolegend, CAT# 423114) following manufacturer's instructions for 20 min at RT and in the dark. Cells were subsequently washed in FACS Buffer (PBS, 1 % BSA) and stained using APC anti-B2M Ab (Biolegend, 316312, 1 in 100 FACS buffer) and PE anti-FLAG Ab (Biolegend, 637310, 1 in 100 FACS buffer) for 30 mins at 4 degrees and in the dark. Cells were washed again in FACS buffer, resuspended in 300 ul FACS buffer and acquired.

TRE	Commercial 1X sequence	Restriction sites used	Origin	Destination
**NFAT-RE**	GGAGGAAAAACTGTTTCATACAGAAGGCGT GGAGGAAAAACTGTTTCATACAGAAGGCGT GGAGGAAAAACTGTTTCATACAGAAGGCGT	NheI HindIII	pNL[NFAT-RE-NLuc-PEST-Hygro] (Promega, UK)	pNFAT1X-NLucP (Twist vector + NFAT)
**NfkB**	CGCATGCATGAGGGGAATTTCCGGGGACTTTCCCGGGAATTT GCGGGGGACTTTCCGGGAATTTCCTCCCTCGAGGATATCAAA TCTGGCCTCGGCGGC	N/A	pNfkB1X-RedFP (Twist vector)	N/A
**SRE**	AGGATGTCCATATTAGGACATCTAGGATGTCCATATTAGGACATCT AGGATGTCCATATTAGGACATCTAGGATGTCCATATTAGGACATCT AGGATGTCCATATTAGGACATCT	NheI HindIII	pNL[SRE-NLuc-PEST-Hygro] (Promega, UK)	pSRE1X-RLucP (Twist vector + SRE)
**SRF**	GTCCATATTAGGACATCTACCATGTCCATATTAGGACATCTACCAT GTCCATATTAGGACATCTACCATGTCCATATTAGGACATCTACCAT GTCCATATTAGGACATCTACCAT	NheI HindIII	pNL[SRF-RE-NLuc-PEST-Hygro] (Promega, UK)	pSRF1X-GrRLucP (Twist vector + SRE)
**CRE**	GCACCAGACAG**TGACGTCA**GCTGCCAGATCCCATGGCCGTCATACT GTGACGTCTTTCAGACACCCCAT**TGACGTCA**ATGGGAGAACAGATCT	NheI HindIII	pNL[CRE-NLuc-PEST-Hygro] (Promega, UK)	pCRE1X-FLucP (Twist vector + SRE)

Supplementary Table 1. Table to show TRE sequences of all TREs used. Each TRE was cloned from its origin vector into its destination vector using the restriction sites. This was not the case for NfkB as the TRE was ordered within the construct from Twist bio.

TRE	Forward primer sequence introducing restriction site	Reverse primer sequence
**NFAT-RE**	GGAGCGGGATCCGGAGGAAAAACTGTTTCATACAGAAGGCGTGGAGG	AAGTCGAGCTTCCATTATATACCCTCTA
**SRE**	GCCGGGGATCCAGGATGTCCATATTAGGACATCTAGGATGT	AAGTCGAGCTTCCATTATATACCCTCTA
**SRF-RE**	GGCCGGGGATCCAGTATGTCCATATTAGGACATCTACCATGTCCATATTAGG	AAGTCGAGCTTCCATTATATACCCTCTA
**NfkB**	CGGCAAGCTTCCGCATGCATGAGGGGAATTT	AAGTCGAGCTTCCATTATATACCCTCTA
**CRE**	GGGCGAAGCTTGCACCAGACAGTGACGTCAGCTGCCAGATCCCAT	AAGTCGAGCTTCCATTATATACCCTCTA

Supplementary Table 2. Table to show primer sequences to add *BamH*I or *Hind*III sites at the 5’ end. These primers were used to amplify TRE regions before concatenation.

Cell-type	Transfection reagent	GPCR	Reporter construct	GPCR mass (ng/well)	Reporter mass (ng/well)	pcDNA3.1 mass (ng/well)	Total mass per well (6-well)
HEK293T	Lipofectamine 2000	M_3R	NFAT1X-NLucP<br>NFAT2X-NLucP<br>NFAT3X-NLucP<br>NFAT4X-NLucP	1	1000	999	2000
HEK293T	Lipofectamine 2000	H_1R	NFAT1X-NLucP<br>NFAT2X-NLucP<br>NFAT3X-NLucP<br>NFAT4X-NLucP	100	1000	900	2000
HEK293T	Lipofectamine 2000	OTR	NFAT1X-NLucP<br>NFAT2X-NLucP<br>NFAT3X-NLucP<br>NFAT4X-NLucP	10	1000	990	2000
HEK293T	Lipofectamine 2000	PTHR1	NfKB1X-RedFP<br>NfKB2X-RedFP<br>NfKB3X-RedFP	100	1000	900	2000
HEK293T-P2YR12	Lipofectamine 2000	Stable cell line	SRF1X-NLucP<br>SRF2X-NLucP<br>SRF3X-NLucP<br>SRF4X-NLucP	0	2000	0	2000
HEK293T-P2YR12	Lipofectamine 2000	Stable cell-line	SRE1X-NLucP<br>SRE2X-NLucP<br>SRE3X-NLucP<br>SRE4X-NLucP	0	2000	0	2000
HEK293T	Lipofectamine 2000	GIPR	NFAT1X-NLucP<br>NFAT2X-NLucP<br>NFAT3X-NLucP<br>NFAT4X-NLucP	100	1000	900	2000
HEK293T	Lipofectamine 2000	GLP-1R	NFAT1X-NLucP<br>NFAT2X-NLucP<br>NFAT3X-NLucP<br>NFAT4X-NLucP	100	1000	900	2000
HEK293T	Lipofectamine 2000	PTHR1	NFAT1X-NLucP<br>NFAT2X-NLucP<br>NFAT3X-NLucP<br>NFAT4X-NLucP	100	1000	900	2000
HEK293T	Lipofectamine 2000	CB1	NFAT1X-NLucP<br>NFAT2X-NLucP<br>NFAT3X-NLucP<br>NFAT4X-NLucP	100	1000	900	2000
HEK293	Transit LT	None	NFAT1X-NLucP<br>NFAT2X-NLucP<br>NFAT3X-NLucP<br>NFAT4X-NLucP	0	1000	1000	2000
HeLa	Lipofectamine 2000	None	NFAT1X-NLucP<br>NFAT2X-NLucP<br>NFAT3X-NLucP<br>NFAT4X-NLucP	0	2000	0	2000
A-549	Transit LT	None	NFAT1X-NLucP<br>NFAT2X-NLucP<br>NFAT3X-NLucP<br>NFAT4X-NLucP	0	5000 (T25 flask)	0	5000 (T25 flask)

Supplementary Table 3. Table to show transfection conditions for each experiment. These DNA masses per well of a 6 well or per T25 flask were used for the data displayed.

Step	Temperature	Time	Cycle
Initial denaturation	98	30 sec	1
Denaturation	98	10 sec	40
Annealing	55-65	10 sec	
Extension	72	60 sec (30 s/ Kb)	
Final extension	72	5 min	1
Hold	4	~	~

Supplementary Table 4. Table to PCR cycling conditions for HDRT generation gradient PCR.

Cell-type	Rounded KI efficiency (%)	Viability (%)	Live count cell count (1 x 10^6 cells/ µl)	Final density FLAG-CD52+ cells (cells per ml)	Final density total (cells per ml)	Number of cells assayed in 500 µl (FLAG-CD52+/Total)
**NFAT 1X-KI**	29	95.9	2.1	290,000	1,000,000	145,000/500,000
**NFAT 2X-KI**	21	95.2	2.3	290,000	1,380,952	145,000/690,476
**NFAT3X-KI**	24	95.3	2.6	290,000	1,208,333	145,000/604,167
**NFAT4X-KI**	15	95.5	2.1	290,000	1,933,333	145,000/966,667

Supplementary Table 5. Calculation of seeding densities to maintain equal number of FLAG-CD52+ cells across NFAT 1X-KI, NFAT 2X-KI, NFAT 3X-KI, and NFAT 4X-KI populations of T cells in final assay.
