# Supplementary material for: Enhancement of a nuclear factor of activated T cells (NFAT) reporter for the study of G protein-coupled receptors
Source: Commun Biol. 2026 Apr 26;9:882. doi: 10.1038/s42003-026-10110-5 (PMC13323705; doi:10.1038/s42003-026-10110-5)
Supplement: Supplementary file 2 — Description of Additional Supplementary Files [file 42003_2026_10110_MOESM2_ESM.docx]

Description of Additional Supplementary File

File name: Supplementary Data 1
Description: Data set for Figure 2

File name: Supplementary Data 2
Description: Data set for Figure 3

File name: Supplementary Data 3
Description: Data set for Figure 4

File name: Supplementary Data 4
Description: Data set for Figure 5

File name: Supplementary Data 5
Description: Data set for Figure 6

File name: Supplementary Data 6
Description: Data sets for the figures in the Supplementary data
