# Supplementary material for: Enhancement of a nuclear factor of activated T cells (NFAT) reporter for the study of G protein-coupled receptors
Source: Commun Biol. 2026 Apr 26;9:882. doi: 10.1038/s42003-026-10110-5 (PMC13323705; doi:10.1038/s42003-026-10110-5)
Supplement: Supplementary file 9 — Reporting Summary [file 42003_2026_10110_MOESM9_ESM.pdf]

nature portfolio

Corresponding author(s): Graham Ladds

Last updated by author(s): Apr 25, 2025

Reporting Summary

Nature Portfolio wishes to improve the reproducibility of the work that we publish. This form provides structure for consistency and transparency in reporting. For further information on Nature Portfolio policies, see our Editorial Policies and the Editorial Policy Checklist.

Statistics

For all statistical analyses, confirm that the following items are present in the figure legend, table legend, main text, or Methods section.

n/a	Confirmed	
☐	☒	The exact sample size (*n*) for each experimental group/condition, given as a discrete number and unit of measurement
☐	☒	A statement on whether measurements were taken from distinct samples or whether the same sample was measured repeatedly
☐	☒	The statistical test(s) used AND whether they are one- or two-sided<br>*Only common tests should be described solely by name; describe more complex techniques in the Methods section.*
☒	☐	A description of all covariates tested
☐	☒	A description of any assumptions or corrections, such as tests of normality and adjustment for multiple comparisons
☐	☒	A full description of the statistical parameters including central tendency (e.g. means) or other basic estimates (e.g. regression coefficient) AND variation (e.g. standard deviation) or associated estimates of uncertainty (e.g. confidence intervals)
☐	☒	For null hypothesis testing, the test statistic (e.g. *F*, *t*, *r*) with confidence intervals, effect sizes, degrees of freedom and *P* value noted<br>*Give P values as exact values whenever suitable.*
☒	☐	For Bayesian analysis, information on the choice of priors and Markov chain Monte Carlo settings
☒	☐	For hierarchical and complex designs, identification of the appropriate level for tests and full reporting of outcomes
☒	☐	Estimates of effect sizes (e.g. Cohen's *d*, Pearson's *r*), indicating how they were calculated

Our web collection on statistics for biologists contains articles on many of the points above.

Software and code

Policy information about availability of computer code

Data collection	Data was collected using SMART control CLARIOstar Plus exported to Excel files using MARS v4.2. Software for the electrophoresis gels? Anything else?
Data analysis	Data was analysed using Graphpad prism 10.4.1 for three parameter fitting of dose response data.

For manuscripts utilizing custom algorithms or software that are central to the research but not yet described in published literature, software must be made available to editors and reviewers. We strongly encourage code deposition in a community repository (e.g. GitHub). See the Nature Portfolio guidelines for submitting code & software for further information.

Data

Policy information about availability of data

All manuscripts must include a data availability statement. This statement should provide the following information, where applicable:

- Accession codes, unique identifiers, or web links for publicly available datasets
- A description of any restrictions on data availability
- For clinical datasets or third party data, please ensure that the statement adheres to our policy

Data Availability: Source data are provided with this paper as supplementary files.

Research involving human participants, their data, or biological material

Policy information about studies with human participants or human data. See also policy information about sex, gender (identity/presentation), and sexual orientation and race, ethnicity and racism.

Reporting on sex and gender	N/A
Reporting on race, ethnicity, or other socially relevant groupings	N/A
Population characteristics	N/A
Recruitment	N/A
Ethics oversight	N/A

Note that full information on the approval of the study protocol must also be provided in the manuscript.

Field-specific reporting

Please select the one below that is the best fit for your research. If you are not sure, read the appropriate sections before making your selection.

☒ Life sciences ☐ Behavioural & social sciences ☐ Ecological, evolutionary & environmental sciences

For a reference copy of the document with all sections, see nature.com/documents/nr-reporting-summary-flat.pdf

Life sciences study design

All studies must disclose on these points even when the disclosure is negative.

Sample size	All sample sizes were determined using the guidelines from the British Journal of Pharmacology (instructions to authors and the published guidelines editorial (Curtis M et al Br J Pharmacol. 2018 Apr;175(7):987-993).
Data exclusions	All data analysis adhered to the guidelines described in - Curtis M et al Br J Pharmacol. 2018 Apr;175(7):987-993. Data exclusion was not performed except for instances where control experiments failed and then the entire experiment was removed from analysis.
Replication	When using primary cell lines, there is a degree of variation in the precise potency and extend of signalling magnitude. All experiments were appropriately controlled using "system pathway agonists". SAll experiments were appropriately controlled using 'system pathway' agonists. If any of these pathway controls generated inappropriate responses, then the entire data set was removed from analysis if not shown to be experimental error.
Randomization	Randomization in this study was not performed due to the limited number of reagents and cell lines used. All data generated is entirely novel and signalling pathways not previously explored. As such there was no "preconception" of what "should have happened". To ensure robustness of data, independent members of the research team preformed some overlapping experiments to determine reproducibility. On occasion the experimentalist was not informed of the compounds used or cells lines investigated.
Blinding	See comments on randomization regarding some aspects of blinding.

Reporting for specific materials, systems and methods

We require information from authors about some types of materials, experimental systems and methods used in many studies. Here, indicate whether each material, system or method listed is relevant to your study. If you are not sure if a list item applies to your research, read the appropriate section before selecting a response.

Materials & experimental systems

n/a	Involved in the study	
☐	☒	Antibodies
☐	☒	Eukaryotic cell lines
☒	☐	Palaeontology and archaeology
☒	☐	Animals and other organisms
☒	☐	Clinical data
☒	☐	Dual use research of concern
☒	☐	Plants

Methods

n/a	Involved in the study	
☒	☐	ChIP-seq
☐	☒	Flow cytometry
☒	☐	MRI-based neuroimaging

Antibodies

Antibodies used	Antibodies used for flow cytometry were APC anti-B2M Ab (Biolegend, CAT#316312) and PE anti-FLAG Ab (Biolegend, CAT#637310)
Validation	Data showing validation in flow cytometry for each antibody is available on the Biolegend website.

Eukaryotic cell lines

Policy information about cell lines and Sex and Gender in Research

Cell line source(s)	HEK293 and HEK293T cells were obtained from ATCC. HeLa cell lines were from ATCC. A549 cells were obtained from were the AstraZeneca Global Cell Bank.
Authentication	Authentication: HEK293T cells from ATCC were validated as described in https://www.atcc.org/products/crl-3216. Short tandem repeat profiling was used to authenticate HeLa cells (Eurofins, Germany) and HEK293 cells (Public Health England) (https://www.nature.com/articles/s41467-017-01644-8). A549 cells were authenticated by suppliers.
Mycoplasma contamination	All cell lines that enter the lab are firstly tested for microplasma contamination. All passed.
Commonly misidentified lines (See ICLAC register)	None of the cell lines used are listed in v12 of the ICLAC register.

Plants

Seed stocks	N/A
Novel plant genotypes	N/A
Authentication	N/A

Flow Cytometry

Plots

Confirm that:

☒ The axis labels state the marker and fluorochrome used (e.g. CD4-FITC).

☒ The axis scales are clearly visible. Include numbers along axes only for bottom left plot of group (a 'group' is an analysis of identical markers).

☒ All plots are contour plots with outliers or pseudocolor plots.

☒ A numerical value for number of cells or percentage (with statistics) is provided.

Methodology

Sample preparation	~1.5 x 10^5 cells were pelleted (300 x g for 5 min). Cells were washed once in PBS and stained with Zombie Violet™ Fixable Viability Kit (Biolegend, CAT# 423114) following manufacturer's instructions for 20 min at RT and in the dark. Cells were subsequently washed in FACS Buffer (PBS, 1 % BSA) and stained using APC anti-B2M Ab (Biolegend, 316312, 1 in 100 FACS buffer) and PE anti-FLAG Ab (Biolegend, 637310, 1 in 100 FACS buffer) for 30 mins at 4 degrees and in the dark. Cells were washed again in FACS buffer, resuspended in 300 ul FACS buffer and acquired as below.
Instrument	Cells were acquired on the BD LSR-Fortessa Cell Analyzer (BD Bioscience).
Software	DB FACS Diva was used for acquisition. BD FlowJO was used for data analysis as per gating strategy below.
Cell population abundance	Percentages of populations relevant to this analysis (Live/ lymphocyte/ single cell/ iCasp9-CD19+ TCRαβ-) were determine as per the gating strategy below.

Gating strategy

(1) Live cell gate (2) Lymphocyte gate (3) Doublet exclusion gate (4) % FLAG-CD-52+/ B2M- (B2M/FLAG-CD52+ KI cells).

☒ Tick this box to confirm that a figure exemplifying the gating strategy is provided in the Supplementary Information.
